# Supplementary material for: Acute exercise as active inference in chronic musculoskeletal pain, effects on gait kinematics and muscular activity in patients and healthy participants: a study protocol for a randomised controlled laboratory trial
Source: BMJ Open. 2023 May 31;13(5):e069747. doi: 10.1136/bmjopen-2022-069747 (PMC10255138; doi:10.1136/bmjopen-2022-069747)
Supplement: Supplementary data [file bmjopen-2022-069747supp002.pdf]

HÖGSKOLAN DALARNA

KOD __________

Samtycke till att delta i projektet

Jag har fått muntlig och skriftlig informationen om projektet och har haft möjlighet att ställa frågor. Jag får behålla den skriftliga informationen.

☐ Jag samtycker till att delta i projektet *Fysisk aktivitet, rörelsekontroll och muskelaktivitet hos personer med långvarig smärta.*

☐ Jag samtycker till att uppgifter om mig behandlas på det sätt som beskrivs i forskningspersonsinformationen.

Ort och datum	Underskrift

Högskolan Dalarna EPN-nr 2018-307

S 791 88 Falun | Tfn +46 23-77 80 00 | Fax +46 23-77 80 80 | www.du.se

Sweden

HÖGSKOLAN DALARNA

KOD __________

Forskningspersonens kopia

Samtycke till att delta i projektet

Jag har fått muntlig och skriftlig informationen om projektet och har haft möjlighet att ställa frågor. Jag får behålla den skriftliga informationen.

☐ Jag samtycker till att delta i projektet *Fysisk aktivitet, rörelsekontroll och muskelaktivitet hos personer med långvarig smärta.*

☐ Jag samtycker till att uppgifter om mig behandlas på det sätt som beskrivs i forskningspersonsinformationen.

Ort och datum	Underskrift

Westergren J, *et al. BMJ Open* 2024; 13:e069747. doi: 10.1136/bmjopen-2022-069747

FYSISK AKTIVITET VID LÅNGVARIG SMÄRTA

HÖGSKOLAN DALARNA

Vill du vara med i en forskningsstudie?

Projektet har som syfte att förbättra rehabiliteringen för personer med långvarig smärta.

Långvarig smärta i leder och muskler leder till fysisk och psykisk ohälsa. En viktig del av behandlingen är fysisk träning som anpassas till individens förutsättningar.

Moderna aktivitetsmätare som ser ut som vanliga armbandsklockor blir alltmer vanliga i forskning och utveckling som rör fysisk aktivitet och träning för olika patientgrupper, men få sådana studier har genomförts bland personer med långvarig smärta.

Befintlig forskning har visat att en del personer som lever med långvarig smärta har förändrat rörelsemönster och muskelaktivering, men återigen finns det få studier visar hur fysisk aktivitet och träning påverkar sådana avvikande rörelsebeteenden. För att få sådan vetenskaplig kunskap behövs det mätningar på både personer som lever med långvarig smärta men även mätningar på friska smärtfria personer.

Vad innebär det att delta i projektet?

Du kommer först att bli kontaktad via telefon där du får mer information om projektet. Om du då är intresserad att delta som forskningsperson och matchar projektets urvalskriterier (ålder, typ av smärta med mera), bokas ett besök in vid Högskolan Dalarnas idrotts- och hälsolaboratorium på Lugnet i Falun.

Under besöket som beräknas ta cirka tre timmar får du:

1. Besvara två frågeformulär med frågor om ditt nuvarande hälsotillstånd, din bakgrund (ålder, yrke etc.) och frågor som berör smärta.
2. Bära flera mindre mätare som registrerar rörelsemönster, muskelaktivitet och fysisk aktivitet. Du kommer gå en kortare sträcka inomhus under några minuter på tre olika sätt och därefter lottas till att antingen cykla eller vila i 30 minuter. Därefter går du samma sträcka som tidigare. Avslutningsvis kommer du att promenera i 18 minuter på ett gångband samtidigt som din utandningsluft mäts via en ansiktsmask.
3. Efter testdagen får du med dig tre mätare hem för att bära under tre dagar på handleden och vid midjan. Dessa postar du åter i ett frankerat kuvert.

Vad händer med mina uppgifter?

Högskolan Dalarna är forskningshuvudman. All insamlad data kommer att ligga till grund för vetenskapliga publikationer. Datan kommer att kodas och förvaras på Högskolan Dalarnas lösenordskyddade dataserver.

Kontakt

Jens Westergren, Doktorand, Vårdvetenskap, e-post: jws@du.se, telefon: 023-77 80 69

Veronica Sjöberg, Doktorand, Vårdvetenskap, e-post: vsj@du.se , telefon: 023-77 87 57

MER INFORMATION OM PROJEKTET HITTAR DU PÅ DU.SE/PAIN

du.se

EPN 2018-307 & 2019-06110
