# Supplementary material for: Acute exercise as active inference in chronic musculoskeletal pain, effects on gait kinematics and muscular activity in patients and healthy participants: a study protocol for a randomised controlled laboratory trial
Source: BMJ Open. 2023 May 31;13(5):e069747. doi: 10.1136/bmjopen-2022-069747 (PMC10255138; doi:10.1136/bmjopen-2022-069747)
Supplement: Author's manuscript [file bmjopen-2022-069747.draft_revisions.pdf]

BMJ Open is committed to open peer review. As part of this commitment we make the peer review history of every article we publish publicly available.

When an article is published we post the peer reviewers' comments and the authors' responses online. We also post the versions of the paper that were used during peer review. These are the versions that the peer review comments apply to.

The versions of the paper that follow are the versions that were submitted during the peer review process. They are not the versions of record or the final published versions. They should not be cited or distributed as the published version of this manuscript.

BMJ Open is an open access journal and the full, final, typeset and author-corrected version of record of the manuscript is available on our site with no access controls, subscription charges or pay-per-view fees (http://bmjopen.bmj.com).

If you have any questions on BMJ Open's open peer review process please email info.bmjopen@bmj.com

BMJ Open

Acute exercise as active inference in chronic musculoskeletal pain, effects on gait kinematics and muscular activity in patients and healthy participants – A study protocol for a randomised controlled laboratory trial

Journal:	*BMJ Open*
Manuscript ID	bmjopen-2022-069747
Article Type:	Protocol
Date Submitted by the Author:	31-Oct-2022

Complete List of Authors:	Westergren, Jens; Dalarna University, School of Health and Welfare, Department of Sports and Medicine<br>Sjöberg, Veronica; Dalarna University, School of Health and Welfare, Department of Sports and Medicine<br>Vixner, Linda; Hogskolan Dalarna, School of Health and Welfare, Department of Sports and Medicine; Uppsala University, Department of Research and Education, Center for Clinical Research Dalarna, Region Dalarna<br>Nyberg, Roger; Dalarna University, School of Technology and Business Studies<br>Conradsson, David; Karolinska Institutet, Department of Neurobiology, Care Sciences and Society, Division of Physiotherapy; Karolinska Universitetssjukhuset, Medical unit Occupational therapy & Physiotherapy, Theme Women's Health and Allied Health Professional<br>Monnier, Andreas; Dalarna University, School of Health and Welfare, Department of Sports and Medicine; Karolinska Institutet, Department of Neurobiology, Care Sciences and Society, Division of Physiotherapy<br>LoMartire, Riccardo; Uppsala University, Department of Research and Education, Center for Clinical Research Dalarna, Region Dalarna<br>Enthoven, Paul; Linköping University, Department of Health, Medicine and Caring Sciences, Pain and Rehabilitation Centre<br>Äng, Björn; Dalarna University, School of Health and Welfare, Department of Sports and Medicine; Uppsala University, Department of Research and Education, Center for Clinical Research Dalarna, Region Dalarna

Keywords:	Back pain < ORTHOPAEDIC & TRAUMA SURGERY, PAIN MANAGEMENT, Musculoskeletal disorders < ORTHOPAEDIC & TRAUMA SURGERY

SCHOLARONE™
Manuscripts

BMJ Open

Words in text 3995

Words in abstract 286

Tables 1

Figures 3

Appendices 1 (SPIRIT checklist)

Acute exercise as active inference in chronic musculoskeletal pain, effects on gait kinematics and muscular activity in patients and healthy participants – A study protocol for a randomised controlled laboratory trial

Jens Westergren,[1] Veronica Sjöberg,[1] Linda Vixner,[1,6] Roger G Nyberg,[1,7] David Moulaee Conradsson,[2,3] Andreas Monnier,[1,2,8] Riccardo LoMartire,[1,2] Paul Enthoven,[4,5] Björn O Äng.[1,2,6]

[1]Department of Sport and Medicine, School of Health and Welfare, Dalarna University, Falun, Sweden

[2]Department of Neurobiology, Care Sciences and Society, Division of Physiotherapy, Karolinska Institutet, Huddinge, Sweden

[3]Medical unit Occupational therapy & Physiotherapy, Theme Women's Health and Allied Health Professional, Karolinska University Hospital, Stockholm, Sweden

[4]Department of Health, Medicine and Caring Sciences, Physiotherapy, Linköping University, Linköping, Sweden

[5]Pain and Rehabilitation Centre, and Department of Health, Medicine and Caring Sciences, Linköping University, Linköping, Sweden

[6]Department of Research and Education, Center for Clinical Research Dalarna – Uppsala University, Region Dalarna, Falun, Sweden

[7]School of Technology and Business Studies, Dalarna University, Falun, Sweden

[8] Military Academy Karlberg, Swedish Armed Forces, Solna, Sweden

For peer review only

For peer review only - http://bmjopen.bmj.com/site/about/guidelines.xhtml

Corresponding author:
Jens Westergren
Department of Sport and Medicine, School of Health and Welfare, Dalarna University, Falun, Sweden
E-Mail: jws@du.se

For peer review only

ABSTRACT

Introduction

Chronic musculoskeletal pain is a highly prevalent, complex and distressing condition that may negatively affect all domains of life. In view of an active inference framework, and resting on the concept of allostasis, human movement per se becomes a prerequisite for health and wellbeing while chronic pain becomes a sign of a system unable to attenuate an allostatic load. Previous studies on different subgroups of chronic pain conditions have demonstrated alterations in gait kinematics and muscle activity, indicating shared disturbances in the motor system from long-term allostatic load. We hypothesize that such alterations exist in heterogenous populations with chronic musculoskeletal pain, and that exposure to acute and controlled exercise, may attenuate these alterations. Therefore, the main aim of this study is to investigate the acute effects of exercise on gait kinematics and activity of the back and neck muscles during diverse walking conditions in patients with chronic musculoskeletal pain compared to a reference sample consisting of healthy participants.

Methods and analysis

This two-sample two-armed parallel randomised controlled laboratory trial will include 40 participants with chronic musculoskeletal pain (> 3 months) and 40 healthy participants. Participants will be randomly allocated to either 30 min of aerobic exercise or rest. Primary outcomes are gait kinematics (walking speed, step frequency, stride length, lumbar rotation, gait stability) and muscular activity (spatial and temporal) of the back and neck during diverse walking conditions. Secondary outcomes are variability of gait kinematics and muscle activity and subjective pain ratings assessed regularly during the trial.

For peer review only

For peer review only - http://bmjopen.bmj.com/site/about/guidelines.xhtml

Ethics and dissemination

The study has been approved by the Regional Ethics Review Board in Uppsala, Sweden (#2018/307/308). Findings will be disseminated via conference presentations, publications in peer-reviewed journals and engagement with patient support groups and clinicians.

Trial Registration: ClinicalTrials.gov; NCT03882333, March 20, 2019.

Keywords:

Chronic Musculoskeletal Pain, Active Inference, Acute Aerobic Exercise, Gait Kinematics, Electromyography

For peer review only

For peer review only - http://bmjopen.bmj.com/site/about/guidelines.xhtml

Strengths and limitations of this study

- To our knowledge, this will be the first randomised controlled laboratory trial investigating the acute effects of aerobic exercise, as active inference, on gait kinematics and muscular activity in a heterogenous group of patients with chronic musculoskeletal pain.
- As long-term aerobic exercise is ultimately the result of regular sessions of aerobic exercise, studying effects induced by a single session of aerobic exercise will provide important insights into exercise and its role in the rehabilitation of chronic pain conditions.
- The study will incorporate objective measures of gait kinematics and muscle activity from continuous overground walking by utilizing modern wireless sensors, thus replicating real world conditions of functional movement.
- While complete blinding is not possible post intervention, due to the nature of exercise, care will be taken to minimize investigator bias by using automated recording protocols.

Based on protocol version 1.0; May 12, 2020.

For peer review only

For peer review only - http://bmjopen.bmj.com/site/about/guidelines.xhtml

INTRODUCTION

Chronic musculoskeletal pain is a highly prevalent and complex condition that continues to challenge clinicians and academics and as such constitutes a major health and wellbeing burden. [1 2] The International Association for the Study of Pain (IASP) defines chronic pain as "pain which has persisted beyond normal tissue healing time", which, in the absence of other factors, is a pain condition lasting for more than three months. [3] Conditions with chronic musculoskeletal pain may be referred to as chronic primary pain[4] and should be seen as a disease in its own right. [5] It can negatively affect physical movement and functioning, sleep, social interaction, quality of life and work ability, [6] in essence all domains of life.

In the light of an active inference framework, [7-9] based on the free energy principle proposed by Friston[10 11] and resting on the concept of allostasis, [12] any living system becomes a model of the world it inhabits. Then, through the process of active inference it attempts to maximize the proof of its own existence (chances of survival) by minimizing prediction errors (variational free energy) through perception and action. The physiological concept of allostasis can be described as achieving stability through change, [12] in which the rate of change influences the process of adaption. In addition, living things may be defined by their ability to move autonomously, be it of bodies, limbs or cells, making movement a key driver of allostasis through sensory palpation of the world. In view of this, human movement, from cell respiration to locomotion, may constitute the major source of active inference upon which the organism builds and maintains its model, making human movement of all sorts pertinent to long-term health and prosperity by facilitating the changes that are needed for stability.

For peer review only - http://bmjopen.bmj.com/site/about/guidelines.xhtml

Building on this notion, in a healthy context acute pain can be viewed as a precision signal for learning and control, with the main function being motivational to direct both short- and long-term motor behaviour away from harm. [13] Chronic musculoskeletal pain on the other hand, is suggestive of a living system in distress and may be explained by the creation and maintenance of abnormal priors that are afforded abnormal attention in a self-evidencing system. [7 14]

This reasoning is – we believe – in line with earlier theories such as the body-self neuromatrix model, [15] the theory of Moving differently in pain, [16] and effectively aligning with concepts such as sensitization[17 18] and ultimately depression[19] within chronic pain populations, as well as recent theory on active interoceptive inference. [20] Then chronic pain can also be viewed as the consequence of an allostatic overload from unresolved predictions errors of the systems lower level predictions about its internal and external milieu. [17 19 21-24] These prediction errors in lower level predictions may manifest themselves at higher levels as fear avoidance behaviour of movement[25] and intolerance of uncertainty, [26] which lead to the further reduction of physical activity. [27-29] This relative sensory deprivation[30 31] that is induced by sedentary behaviour may disable self-organisation normally driven through allostasis, leading to aberrant neuro-signatures (models) of painful states. In essence, chronic musculoskeletal pain becomes a pathophysiology with a self-fulfilling hypothesis of pain as the systems only solution to minimize prediction errors. Building on this notion, physical activity and exercise, or simply human movement, can be viewed as active inference and as such may facilitate a return to allostasis and less painful future states by promoting the flexible reassignment of precision weighting that alters the individual's prediction about their body and their world. [32] This implies that adding physical activity or exercise to a rehabilitation program, may simply be reintroducing something essential that was engineered away generations ago.

For peer review only - http://bmjopen.bmj.com/site/about/guidelines.xhtml

From this active inference view of chronic pain, it is understandable that a biopsychosocial approach has gained traction in the treatment of chronic pain conditions,[6 33 34 35] since such interdisciplinary treatment delivered by a team of allied health professionals involves a combination of physical activity and exercise, psychological interventions, pharmaceutical treatments, and patient education all acting as active inference[36] enabling a return to less painful states.

Given the fact that the musculoskeletal system is crucial to human locomotion or functional movement, a large body of research has focused on the effects of chronic pain on functional movement. There is now moderate to strong evidence that individuals with persistent low back pain demonstrate differences in gait kinematics,[37] that individuals with non-specific low back pain demonstrate impaired postural control when standing,[38] and that individuals diagnosed with fibromyalgia demonstrate an altered gait compared to healthy individuals.[39] Neuroimaging studies have reported functional brain changes related to sensorimotor impairment in individuals with low back pain,[40] irritable bowel syndrome,[14] fibromyalgia[41] and migraine,[42] suggestive of shared disturbances of the motor system across different diagnoses. The general finding is that fibromyalgia patients walk slower, with a lower step frequency and shorter step-length.[37]

However, based on the notion of a shared disturbance it is currently not known if the altered gait patterns identified in specific sub-groups of chronic pain patients may generalize to heterogeneous populations of chronic musculoskeletal pain patients and if they could be used as markers of functional movement impairment in these populations. There is a paucity in how exercise, viewed as active inference can have an acute effect on the motor control of

For peer review only - http://bmjopen.bmj.com/site/about/guidelines.xhtml

walking in humans, especially in adaptions to perturbations such as faster walking speeds or dual tasks and if this is related to long-term sedentary behaviour.

Therefore, the main aim of this study is to investigate the acute effects of exercise as active inference on gait kinematics and activity of the back and neck muscles during diverse walking conditions in patients with chronic musculoskeletal pain compared to a reference sample consisting of participants without chronic musculoskeletal pain.

Our hypothesis is that a heterogenous sample with chronic musculoskeletal pain will walk at lower speeds, lower step frequency and stride lengths but with increased muscle activity in the back and neck and that these differences will increase with task difficulty and begin to attenuate acutely by exercise.

MATERIALS AND METHODS

Trial design

The study consists of a two-sample two-armed parallel randomised controlled laboratory trial (RCT) that will be conducted at Dalarna University, Sweden (Figure 1). The main sample consists of patients with chronic pain who will be randomised into one of two arms: an intervention group performing acute aerobic exercise (30 min stationary cycling) or a resting control group. The reference sample consists of participants without chronic musculoskeletal pain who will follow a similar RCT procedure, with the purpose being to investigate whether the acute response to exercise is unique for patients with chronic pain as our hypothesis proposes, or if it is just a general effect that would be seen any population. The trial will comply with Consolidated Standards of Reporting Trials (CONSORT) and Standard Protocol Items: Recommendations for Interventional Trials guidelines with Patient Reported Outcomes

For peer review only

(SPIRIT-PRO).[43-46] A completed SPIRIT-PRO checklist for the trial can be found in the supporting information. Data will be collected between January 2019 and May 2021.

Sample size for this multi project data collection was largely based on studies described elsewhere, in addition a power calculation from right side Erector Spinae Longissimus normalized back muscle activity collected from our first 20 participants confirmed a sample size of $n = 40$ for a power of 0.80 for $\alpha = 0.05$ (two sided) to identify a 25% difference between patients and participants without musculoskeletal pain (reported elsewhere).

Please insert Figure 1 here

Participants and setting

This trial includes 80 participants. Patients (n=40) that visit primary and specialist pain clinics in Region Dalarna for evaluation or treatment of chronic musculoskeletal pain (> 3 months), as well as an age and gender matched convenience sample of participants without musculoskeletal pain (n=40) will be simultaneously recruited. Physiotherapists at the clinics will inform patients of the study and those interested in participation will be asked to provide their contact information. All potential participants will be contacted by email and telephone prior to the laboratory visit, to perform a preliminary screening of eligibility criteria and schedule a time for testing.

Eligibility criteria

For peer review only - http://bmjopen.bmj.com/site/about/guidelines.xhtml

Participants aged 18-67 years will be included. Patients will be in assessment and/or treatment of chronic (>3 months) musculoskeletal (neck and/or low back) pain or widespread pain in primary or specialist pain care in Region Dalarna. Exclusion criteria: 1) Any co-existing medical condition that would restrict safely participating in the exercise intervention. 2) Previous spinal surgery, 3) Inability to perform unsupported indoor walking, 4) Inadequate Swedish or/cognitive ability to provide consent and understand information and instructions, 5) Pregnant in second or third trimester or childbirth in the last three months, 6) Allergies to adhesive tape used for sensor application, 7) Major hearing impairment effecting performance of auditory 1-back test, 7) Pain caused by malignancies or systemic diseases, or localized pain in lower extremities only.

Randomisation, allocation concealment and blinding

Participants will be randomly assigned to either the intervention or control group at a 1:1 allocation ratio, using stratified block randomization with a fixed block size of six within the chronic musculoskeletal pain and no chronic pain strata. Allocation information is kept in opaque sealed envelopes that will be opened after baseline measurements to blind investigators and participants until baseline measurements are completed. All data collection will be automated in pre-programmed sequences and participant instructions read verbatim to minimize any investigator bias. Participants will not be informed of the hypotheses of the study, and the researcher performing the data analyses will be blinded to group allocation.

For peer review only - http://bmjopen.bmj.com/site/about/guidelines.xhtml

Interventions

The acute aerobic exercise intervention will consist of one 20-minute cycling interval preceded by a five-minute warm-up and followed by a five-minute cool-down (total duration of 30 minutes) and will be performed on a stationary electromagnetically braked cycle ergometer (Schoberer Rad Messtechnik SRM, Jülich, Germany). Self-selected exercise intensity will be guided to moderate intensity by using Borg Ratings of Perceived Exertion (RPE) scale with a target RPE of 13-15 (somewhat hard/ hard).[47] Borg RPE and self-rated pain on VAS (0-100mm) will be recorded every five-minutes during the exercise session. Power output, cadence, and heart rate will be recorded at 2 Hz. Participant will be able to freely regulate cadence and resistance to maintain the target RPE of 13-15, by means of a 14-increment mechanical gear shifter located on the handlebars. The ergometer is equipped with an upright handlebar, flat pedals and a wide padded saddle with an extra gel cover to minimize sitting discomfort.

Participants allocated to the control groups will rest for the equivalent period of 30 minutes in a self-selected comfortable resting position accompanied by staff.

Outcome measures

The primary outcomes (detailed in table 1) are kinematic measures of human movement and muscle activity that will be measured while participants are walking using the following three

For peer review only - http://bmjopen.bmj.com/site/about/guidelines.xhtml

gait conditions: 1) self-selected habitual gait speed, 2) brisk gait speed, and 3) self-selected habitual gait speed with a concurrent cognitive task. Our primary kinematic outcomes are gait speed, step frequency, stride length, lumbar rotation, and gait stability, while primary outcomes for the muscle activity domain will be mean normalized EMG amplitude averaged over aggregated gait cycles.

Our secondary outcome measures are variability of gait kinematics and muscle activity and self-rated current pain intensity using a horizontal 100-mm line Visual Analogue Scale (VAS) with the statement 'no pain at all' at the extreme left, and 'the worst possible pain' at the extreme right (chronic pain sample only). [48 49]

Table 1. Outcomes and assessments

			Baseline	Intervention	Follow-up
Baseline measures	Variable or assessment	Data Source			
Participant characteristics	Age, sex, body mass index (weight, height), educational level, work status, physical activity.	SAQ+ PT	X		
Pain catastrophizing †	Pain Catastrophizing Scale (PCS)	SAQ	X		
Effects on everyday life †	Multidimensional Pain Inventory (MPI)	SAQ	X		
Primary outcome measures					
**Gait kinematics**<br>- Gait speed (m/s)<br>- Step frequency (steps min^{-1})<br>- Stride length (cm)<br>- Lumbar rotation (°)<br>- Lateral step variability (three consecutive steps, the perpendicular deviation of the middle foot placement from the line connecting the first and the third step)<br><br>**Back and neck muscle activity**<br>- Normalized mean muscle activity of all completed gait cycles per trial<br>- Back and neck normalized muscle activity at each 10 percentile of gait cycle	Gait kinematics with IMU-based sensors and wireless surface EMG, aggregated from minimum 50 gait cycles<br>-Self -selected habitual gait speed<br>- Brisk gait speed‡<br>- Self-selected habitual gait speed with concurrent cognitive task (1-back test) ‡	PT	X		X
Secondary outcome measures					
**Variability of gait kinematics**<br>- Gait speed (m/s)<br>- Step frequency (steps min^{-1})<br>- Stride length (cm)<br>- Lumbar rotation (°)<br>- Lateral step variability	Non-linear analysis of gait kinematics with IMU-based sensors and wireless surface EMG, aggregated from minimum 50 gait cycles<br>-Self -selected habitual gait speed<br>- Brisk gait speed‡	PT	X		X

For peer review only

**Variability of muscle activity**<br>- Normalized mean per trial<br>- Balance of left/right at each 10 percentile of gait cycle<br>- Back and neck normalized during stance and swing phase<br>- Number of muscle activity spikes (>100% normalized per complete gait cycle)	- Self-selected habitual gait speed with concurrent cognitive task (1-back test) ‡				
- Pain intensity†	VAS (0-100)	SAQ	X	X	X

Data source: Self-administered questionnaire (SAQ), physical testing (PT).

VAS, Visual Analogue Scale; administered after every gait test. RPE, Rating of Perceived Exertion; every 5 minutes during the intervention; IMU, Inertial Monitoring Unit; EMG, Electromyography.

† Only measured in patients with chronic musculoskeletal pain

‡ Brisk gait speed and self-selected gait speed with the 1-back test will be performed in random order

For peer review only

For peer review only - http://bmjopen.bmj.com/site/about/guidelines.xhtml

Trial procedure, instruments and data collection

The entire trial procedure at the laboratory is expected to take approximately three hours and will be supervised by two researchers. Upon arrival at the laboratory, eligibility screening, presentation of the study procedures and collection of informed consent will be performed. Participants will complete the following questionnaires before commencing any testing: 1) Participant characteristics (age, sex, educational level, occupational status, pain locations, pain duration) including two validated items with questions about physical activity. [50] Participants with chronic musculoskeletal pain will also complete the following two questionnaires relating to pain experiences: 2) The Pain Catastrophizing Scale (PCS) [51] which contains 13 items regarding patients thoughts and feelings associated with pain, with a total score ranging from 0-52, with higher scores representing higher pain catastrophizing and 3) The West Haven-Yale Multidimensional Pain Inventory (MPI), which is a questionnaire that measures the psychosocial, cognitive, and behavioural effects of chronic pain. [52-55]

A researcher will then record participant body height using a stadiometer (Harpenden Stadiometer, Holtain Limited, Crosswell, UK) and body weight using a weighing scale (Midrics, Sartorius AG, Goettingen, Germany). Participants will be introduced to the 1-back test[56] and listen to a 30-second practice sequence, then two 30-second 1-back baseline tests will be recorded with participants in a seated position. The 1-back test will be performed with a pair of minimally intrusive wireless handsfree on-ear headphones connected via Bluetooth to a PC using Audacity Software v. 2.3.0. Participants will listen to an audio file consisting of a randomized number sequence and will be instructed to respond as quickly and accurately as possible to audio prompts, repeating the digit before the last one in the consecutive sequence during the entire 1-back walking trial. All responses will be recorded.

For peer review only - http://bmjopen.bmj.com/site/about/guidelines.xhtml

After this, seven wireless inertial monitoring unit (IMU) sensors (OPAL, APDM INC, OR, USA) will be applied on participants using elastic straps as specified in figure 2. [57] Gait kinematics will be captured at 128Hz and automatically processed via Moveo Explorer ™ software. Participants will be given standardized instructions prior to starting each gait trial in order to comply with the initial calibration procedure described by the manufacturer.

Please insert Figure 2 here

Wireless electromyography (EMG) sensors (Delsys Trigno, Delsys Inc., Boston, MA, USA) will be positioned as specified in figure 3, in accordance with SENIAM recommendations[58 59] and data recorded at 1111 Hz. Skin will be shaved and cleansed with a chlorhexidine digluconate ethanol cutaneous solution (Klorhexidinsprit 5mg/ml, Fresenius Kabi AB, Uppsala, Sweden). To obtain normalizing (reference) muscle activity, each participant will perform four standardized submaximal standing trunk flexion (hip flexion to 45° from neutral standing position) muscle contraction tests, interspersed by a 60-second rest. EMG sensors will be removed after baseline measurements and reapplied prior to the follow-up walking trials. Before removal sensor positions will be marked with a marker pen to facilitate accurate reapplication.

Please insert Figure 3 here

All gait trials will be performed indoors in a continuous fashion, on a 15-meter standardised walkway with tape markings on the floor indicating starting and 180° turning points. Trials will have a minimum duration of two minutes and finish when participants cross the start line with a time exceeding two minutes. Participants will initially perform a familiarization trial at

For peer review only - http://bmjopen.bmj.com/site/about/guidelines.xhtml

a self-selected habitual speed followed by a short rest if needed, to avoid any accumulated pain or fatigue. After this, participants will proceed to walk the 15-meter walkway under the three different conditions described above: 1) an initial self-selected habitual gait speed, 2) a brisk gait speed and 3) a self-selected habitual gait speed with a concurrent cognitive task (1-back test). The order of performance of the brisk and 1-back walking conditions (2 or 3) will be randomised and the allocation will be kept concealed to the participants until the initial condition with self-selected habitual gait speed has been performed. For the brisk walking condition, participants will be instructed to walk faster than they habitually do, as they imagine themselves as slightly late for a bus they want to catch. The self-rated pain intensity (VAS) of participants with chronic musculoskeletal pain will be recorded after each walk trial. The risk of complication due to measurements or interventions is considered to be low, but any adverse or serious adverse events will be logged and reported.

Data management

Participant questionaries will be checked for completeness and congruity before data is entered into the database. Gait kinematics will be aggregated for all valid gait cycles of each gait condition. For each individual, a minimum of 50 gait cycles will be used and aggregated prior to analysis. [60] Back and neck muscle activity (root-mean square, RMS) synchronized to gait cycles and aggregated from each gait condition will be normalized as percentage RMS against reference contractions.

Statistical analysis

Participant characteristics will be reported using descriptive statistics. The statistical analyses will be performed on an intention to treat basis and potential differences between the intervention group and the control groups. Analysis will primarily be conducted with variance

For peer review only

analysis with respect to both data quality and distribution (data assumptions), and with the addition of repeated measurements where applicable. Log transformations of data will be made as appropriate. Data will be presented with means and variances. To explore the magnitude of the effect of interventions, effect sizes will be calculated.[61] Lastly, non-linear analysis using Gait Evaluation Differential Entropy Method (GEDEM)[62] will be used to assess variability of gait kinematic and muscular activity.[62-64]

Patient and public involvement

The burden of the study procedures has been assessed by patients during preliminary pilot work, primarily to assess the following areas: the feasibility of the cycling exercise intervention, the baseline questionnaires, and the instrumentation to capture gait kinematics and EMG-data. Study participants will be invited to a briefing where the results of the study will be presented. In addition, study participants will be given a summary of the results written in Swedish. Results will be presented at the annual meeting of the Swedish National Registry for Chronic Pain Rehabilitation where patient groups are represented.

DISCUSSION

The current study is to our knowledge the first laboratory RCT to investigate the acute effect of aerobic exercise on gait kinematics and muscle activity in participants with chronic musculoskeletal pain compared to reference participants from a perspective of exercise as active inference. Our two-sample two-armed parallel design allows for a cross comparison of effects in patients and healthy participants that will follow the same study protocol and will contribute to our understanding of how chronic musculoskeletal pain manifest on measures of human motor behaviour and how exercise can influence this in a rehabilitation setting.

For peer review only

For peer review only - http://bmjopen.bmj.com/site/about/guidelines.xhtml

Knowledge of how gait kinematics and back and neck muscular activity may change during different gait conditions and from aerobic exercise may contribute to the understanding of how exercise therapy influences our generative models in the process attenuates allostatic load through active inference. The use of modern, miniaturized, and completely wireless sensors during overground walking replicates real world conditions, hence strengthening the ecological validity of our results. Objective measurements of movement and muscular activity during diverse walking conditions have been chosen as primary outcomes since such a methodology can both uniquely and sensitively show and quantify deviating activation patterns as validly reflected in every day functional movements and commonly performed physical activities.

Participants

Selection bias is difficult to completely overcome in exercise intervention studies as volunteering participants will likely be positively biased towards exercise in general and motivation to participate in an exercise intervention is also likely influenced by the severity of symptoms experienced when asked to volunteer for the study. We will compare and validate our sample baseline characteristics with a new nationwide large database including 60,000 patients with chronic pain. [65]

Concerning our reference participants without chronic musculoskeletal pain, we aim to create a sample that is sex and age matched with our chronic musculoskeletal pain sample. It will serve as a reference sample for all our outcomes – can our hypothesis on the acute effects of exercise in our sample with chronic pain be verified or may unexpected results emerge when healthy participants are subjected to the same intervention?

For peer review only

For peer review only - http://bmjopen.bmj.com/site/about/guidelines.xhtml

Intervention

Viewing this exercise intervention as active inference will hopefully add knowledge on how acute movement through exercise may promote an individual's predictions about their body and their world by creating a platform for belief updating. Our use of a cycling modality with a rather different muscle recruitment and joint loading pattern compared to walking may be beneficial, especially since stationary cycling may be more palatable to participants than running at the same intensity as it may lead to an undesired level of fatigue, fear, or increased pain.

In addition, the aerobic exercise intervention in the current study reflects recommendations in exercise guidelines and allows an easy progression of rehabilitation in terms of both intensity and duration. [66] Since a bicycle can be used for therapy, recreation, and transportation, there are opportunities for health care professionals to facilitate changes in lifestyle that have the potential to mitigate morbidity and function in patients with chronic pain, [67] simultaneously reducing the risks and impact of common co-morbidities.

Outcomes

With the foundation of this project being objective measures of overground walking, it adheres well to the Initiative on Methods, Measurement and Pain Assessment in Clinical Trials (IMMPACT) which recommends that all chronic-pain trials should include measures of physical functioning. [49] This project is expected to result in increased knowledge of how acute exercise effects subsequent functional tasks, here measured during different walking conditions.

For peer review only - http://bmjopen.bmj.com/site/about/guidelines.xhtml

For peer review only

Potential impact

Our proposed project will deepen our understanding on the topic of alterations in functional movement in chronic musculoskeletal pain conditions.

Perspective

Results from the current study may help us reach a better understanding how acute aerobic exercise acts as active inference to attenuate chronic pain by facilitating allostasis. This knowledge will help us formulate better questions as to how generative models in chronic pain conditions are formed, maintained, and updated through movement, or lack thereof.

Conclusion

The results of this dual-sample two-armed parallel randomised controlled laboratory trial with the additional potential for cross-sectional analysis of baseline characteristics will improve our understanding on how acute exercise may or may not influence gait kinematics and muscular activity in participants with chronic musculoskeletal pain. It will hopefully pave a road for further research into human movement as active inference and attenuator of allostatic load. By using modern, minimalistic, wireless sensors during overground walking the study will contribute with new knowledge on the acute effects of aerobic exercise on gait kinematics and muscle activity of the back and neck muscles in patients with chronic pain.

For peer review only - http://bmjopen.bmj.com/site/about/guidelines.xhtml

Author Contributions All authors meet the ICMJE criteria for authorship. JW, VS, BÄ and LV take responsibility for the integrity of the study. All authors were involved in the study´s concept and design. JW and VS will be involved in acquisition of data. JW, PE, and BÄ were involved in drafting and revising the article critically for important intellectual content. All authors were involved in manuscript revision and read and approved the final version to be published.

Funding

This work is supported by the Swedish Research Council for Health (2015-02512), Working life and Welfare (FORTE; 2017-00177), and Dalarna University (N/A), Falun, Sweden. The funders had no role in study design and will have no role in any part of the study or reporting of the results.

Competing interests

None declared.

Patient consent for publication

Not required.

Ethics approval

The study has been approved by the Regional Ethics Review Board in Uppsala, Sweden; approval ID 2018/307/308.

Data sharing statement

Not applicable

For peer review only - http://bmjopen.bmj.com/site/about/guidelines.xhtml

Open access

This is an open access article distributed in accordance with the Creative Commons Attribution Non-Commercial (CC BY-NC 4.0) license, which permits others to distribute, remix, adapt, build upon this work non-commercially, and license their derivative works on different terms, provided the original work is properly cited, appropriate credit is given, any changes made indicated, and the use is non-commercial.

See: http://creativecommons.org/licenses/by-nc/4.0/.

FIGURE CAPTIONS:

Fig. 1. Participant flowchart.
PCS, Pain Catastrophizing Scale; MPI, Multidimensional Pain Inventory.

Figure 2. IMU-sensor placement on the forehead, sternum, lumbar spine, wrists, and feet. IMU, Inertial monitoring unit. Adapted with permission by APDM, INC.

Figure 3. Overview wireless electrode placement for surface EMG recording of the trunk and neck musculature according to SENIAM guidelines (http://www.seniam.org/)

For peer review only

For peer review only - http://bmjopen.bmj.com/site/about/guidelines.xhtml

REFERENCES

1. Leadley RM, Armstrong N, Lee YC, et al. Chronic diseases in the European Union: the prevalence and health cost implications of chronic pain. *Journal of pain & palliative care pharmacotherapy* 2012;26(4):310-25. doi: 10.3109/15360288.2012.736933 [published Online First: 2012/12/12]
2. Global Burden of Disease Study Collaborators. Global, regional, and national incidence, prevalence, and years lived with disability for 328 diseases and injuries for 195 countries, 1990-2016: a systematic analysis for the Global Burden of Disease Study 2016. *Lancet* 2017;390(10100):1211-59. doi: 10.1016/S0140-6736(17)32154-2 [published Online First: 2017/09/19]
3. Raja SN, Carr DB, Cohen M, et al. The revised International Association for the Study of Pain definition of pain: concepts, challenges, and compromises. *Pain* 2020;161(9):1976-82. doi: 10.1097/j.pain.0000000000001939 [published Online First: 2020/07/23]
4. Nicholas M, Vlaeyen JWS, Rief W, et al. The IASP classification of chronic pain for ICD-11: chronic primary pain. *Pain* 2019;160(1):28-37. doi: 10.1097/j.pain.0000000000001390 [published Online First: 2018/12/27]
5. Niv D, Devor M. Chronic pain as a disease in its own right. *Pain Pract* 2004;4(3):179-81. doi: 10.1111/j.1533-2500.2004.04301.x
6. Ahlberg M. Rehabilitering vid långvarig smärta: En systematisk litteraturöversikt (SBU; Swedish agency for health technology assessment and assessment of social services). 2010
7. De Ridder D, Adhia D, Vanneste S. The anatomy of pain and suffering in the brain and its clinical implications. *Neuroscience & Biobehavioral Reviews* 2021;130:125-46. doi: https://doi.org/10.1016/j.neubiorev.2021.08.013
8. Ongaro G, Kaptchuk TJ. Symptom perception, placebo effects, and the Bayesian brain. *Pain* 2019;160(1):1-4. doi: 10.1097/j.pain.0000000000001367
9. Pezzulo G, Parr T, Friston K. The evolution of brain architectures for predictive coding and active inference. *Philos Trans R Soc Lond B Biol Sci* 2022;377(1844):20200531. doi: 10.1098/rstb.2020.0531 [published Online First: 20211227]
10. Friston K. The history of the future of the Bayesian brain. *Neuroimage* 2012;62(2):1230-3. doi: 10.1016/j.neuroimage.2011.10.004 [published Online First: 2011/10/26]
11. Friston K. Life as we know it. *J R Soc Interface* 2013;10(86):20130475. doi: 10.1098/rsif.2013.0475 [published Online First: 20130703]
12. Sterling P, Eyer J. Handbook of Life Stress, Cognition and Health Edited by S. Fisher and J. Reason Copyright 1988 John Wiley 8. Sons, 1988.
13. Seymour B. Pain: A Precision Signal for Reinforcement Learning and Control. *Neuron* 2019;101(6):1029-41. doi: 10.1016/j.neuron.2019.01.055
14. Nisticò V, Rossi RE, D'Arrigo AM, et al. Functional neuroimaging in Irritable Bowel Syndrome: a systematic review highlights common brain alterations with Functional Movement Disorders. *J Neurogastroenterol Motil* 2022 doi: 10.5056/jnm21079 [published Online First: 20220222]
15. Melzack R, Katz J. Pain. *Wiley Interdisciplinary Reviews: Cognitive Science* 2013;4(1):1-15. doi: 10.1002/wcs.1201

For peer review only - http://bmjopen.bmj.com/site/about/guidelines.xhtml

16. Hodges PW, Tucker K. Moving differently in pain: a new theory to explain the adaptation to pain. *Pain* 2011;152(3 Suppl):S90-8. doi: 10.1016/j.pain.2010.10.020 [published Online First: 2010/11/23]
17. Wallden M, Nijs J. Before & beyond the pain – Allostatic load, central sensitivity and their role in health and function. *Journal of Bodywork and Movement Therapies* 2021;27:388-92. doi: https://doi.org/10.1016/j.jbmt.2021.04.003
18. Wallden M, Nijs J. Applying the understanding of central sensitization in practice. *Journal of Bodywork and Movement Therapies* 2021;27:723-30. doi: 10.1016/j.jbmt.2021.04.004
19. Arnaldo I, Corcoran AW, Friston KJ, et al. Stress and its sequelae: An active inference account of the etiological pathway from allostatic overload to depression. *Neuroscience and biobehavioral reviews* 2022;135:104590. doi: 10.1016/j.neubiorev.2022.104590 [published Online First: 20220218]
20. Fermin ASR, Friston K, Yamawaki S. An insula hierarchical network architecture for active interoceptive inference. *R Soc Open Sci* 2022;9(6):220226. doi: 10.1098/rsos.220226 [published Online First: 20220629]
21. Craig AD. How do you feel? Interoception: the sense of the physiological condition of the body. *Nature Reviews Neuroscience* 2002;3(8):655-66. doi: 10.1038/nrn894
22. Craig AD. A new view of pain as a homeostatic emotion. *Trends Neurosci* 2003;26(6):303-7. doi: 10.1016/s0166-2236(03)00123-1 [published Online First: 2003/06/12]
23. Wallden M, Sisson M. Modern disintegration and primal connectivity. *Journal of Bodywork and Movement Therapies* 2019;23(2):359-65. doi: 10.1016/j.jbmt.2019.03.003
24. McEwen BS. Protective and damaging effects of stress mediators: central role of the brain. *Dialogues Clin Neurosci* 2006;8(4):367-81. doi: 10.31887/DCNS.2006.8.4/bmcewen
25. Ellingsen DM, Napadow V, Protsenko E, et al. Brain Mechanisms of Anticipated Painful Movements and Their Modulation by Manual Therapy in Chronic Low Back Pain. *J Pain* 2018;19(11):1352-65. doi: 10.1016/j.jpain.2018.05.012 [published Online First: 20180703]
26. López-Martínez AE, Ramírez-Maestre C, Serrano-Ibáñez ER, et al. Intolerance of uncertainty moderates the relationship between catastrophizing, anxiety, and perceived pain in people with chronic nononcological pain. *Pain Med* 2022 doi: 10.1093/pm/pnac030 [published Online First: 20220216]
27. Parker R, Bergman E, Mntambo A, et al. Levels of physical activity in people with chronic pain. *S Afr J Physiother* 2017;73(1):323. doi: 10.4102/sajp.v73i1.323 [published Online First: 20170331]
28. McBeth J, Nicholl BI, Cordingley L, et al. Chronic widespread pain predicts physical inactivity: results from the prospective EPIFUND study. *European journal of pain (London, England)* 2010;14(9):972-9. doi: 10.1016/j.ejpain.2010.03.005 [published Online First: 2010/04/20]
29. Jones J, Rutledge DN, Jones KD, et al. Self-assessed physical function levels of women with fibromyalgia: a national survey. *Womens Health Issues* 2008;18(5):406-12. doi: 10.1016/j.whi.2008.04.005 [published Online First: 20080823]
30. Blitz B, Lowenthal M. The role of sensory restriction in problems with chronic pain. *J Chronic Dis* 1966;19(11):1119-25.

For peer review only - http://bmjopen.bmj.com/site/about/guidelines.xhtml

31. Mohan A, Vanneste S. Adaptive and maladaptive neural compensatory consequences of sensory deprivation-From a phantom percept perspective. *Prog Neurobiol* 2017;153:1-17. doi: 10.1016/j.pneurobio.2017.03.010 [published Online First: 20170411]
32. Tabor A, Burr C. Bayesian Learning Models of Pain: A Call to Action. *Current Opinion in Behavioral Sciences* 2019;26:54-61. doi: https://doi.org/10.1016/j.cobeha.2018.10.006
33. Kim J, Esteves JE, Cerritelli F, et al. An Active Inference Account of Touch and Verbal Communication in Therapy. *Front Psychol* 2022;13:828952. doi: 10.3389/fpsyg.2022.828952 [published Online First: 20220520]
34. Nicholas M. Task Force on Multimodal Pain Treatment Defines Terms for Chronic Pain Care. cited 2018-02-13. 2017
35. Kamper SJ, Apeldoorn AT, Chiarotto A, et al. Multidisciplinary biopsychosocial rehabilitation for chronic low back pain: Cochrane systematic review and meta-analysis. *BMJ* 2015;350:h444. doi: 10.1136/bmj.h444
36. McParlin Z, Cerritelli F, Friston KJ, et al. Therapeutic Alliance as Active Inference: The Role of Therapeutic Touch and Synchrony. *Frontiers in Psychology* 2022;13 doi: 10.3389/fpsyg.2022.783694
37. Smith JA, Stabbert H, Bagwell JJ, et al. Do people with low back pain walk differently? A systematic review and meta-analysis. *J Sport Health Sci* 2022 doi: 10.1016/j.jshs.2022.02.001 [published Online First: 20220210]
38. Koch C, Hänsel F. Non-specific Low Back Pain and Postural Control During Quiet Standing-A Systematic Review. *Front Psychol* 2019;10:586. doi: 10.3389/fpsyg.2019.00586 [published Online First: 20190322]
39. Carrasco-Vega E, Ruiz-Muñoz M, Cuesta-Vargas A, et al. Individuals with fibromyalgia have a different gait pattern and a reduced walk functional capacity: a systematic review with meta-analysis. *PeerJ* 2022;10:e12908. doi: 10.7717/peerj.12908 [published Online First: 20220321]
40. Goossens N, Rummens S, Janssens L, et al. Association Between Sensorimotor Impairments and Functional Brain Changes in Patients With Low Back Pain: A Critical Review. *Am J Phys Med Rehabil* 2018;97(3):200-11. doi: 10.1097/phm.0000000000000859 [published Online First: 2017/11/08]
41. Cheng JC, Anzolin A, Berry M, et al. Dynamic Functional Brain Connectivity Underlying Temporal Summation of Pain in Fibromyalgia. *Arthritis Rheumatol* 2022;74(4):700-10. doi: 10.1002/art.42013 [published Online First: 20220310]
42. Karsan N, Goadsby PJ. Migraine Is More Than Just Headache: Is the Link to Chronic Fatigue and Mood Disorders Simply Due to Shared Biological Systems? *Front Hum Neurosci* 2021;15:646692. doi: 10.3389/fnhum.2021.646692 [published Online First: 20210603]
43. Calvert M, Kyte D, Mercieca-Bebber R, et al. Guidelines for Inclusion of Patient-Reported Outcomes in Clinical Trial Protocols: The SPIRIT-PRO Extension. *JAMA* 2018;319(5):483-94. doi: 10.1001/jama.2017.21903
44. Chan AW, Tetzlaff JM, Altman DG, et al. SPIRIT 2013 statement: defining standard protocol items for clinical trials. *Ann Intern Med* 2013;158(3):200-7. doi: 10.7326/0003-4819-158-3-201302050-00583 [published Online First: 2013/01/09]

For peer review only - http://bmjopen.bmj.com/site/about/guidelines.xhtml

45. Schulz KF, Altman DG, Moher D. CONSORT 2010 statement: updated guidelines for reporting parallel group randomised trials. *BMJ (Clinical research ed)* 2010;340:c332. doi: 10.1136/bmj.c332 [published Online First: 2010/03/25]
46. Gamble C, Krishan A, Stocken D, et al. Guidelines for the Content of Statistical Analysis Plans in Clinical Trials. *JAMA* 2017;318(23):2337-43. doi: 10.1001/jama.2017.18556 [published Online First: 2017/12/21]
47. Borg G. Perceived exertion as an indicator of somatic stress. *Scand J Rehabil Med* 1970;2(2):92-8.
48. Huskisson EC. Measurement of pain. *Lancet* 1974;2:1127-31.
49. Taylor AM, Phillips K, Patel KV, et al. Assessment of physical function and participation in chronic pain clinical trials: IMMPACT/OMERACT recommendations. *Pain* 2016;157(9):1836-50. doi: 10.1097/j.pain.0000000000000577 [published Online First: 2016/04/09]
50. Brumagne S, Janssens L, Knapen S, et al. Persons with recurrent low back pain exhibit a rigid postural control strategy. *European spine journal : official publication of the European Spine Society, the European Spinal Deformity Society, and the European Section of the Cervical Spine Research Society* 2008;17(9):1177-84. doi: 10.1007/s00586-008-0709-7 [published Online First: 20080702]
51. Sullivan MJL, Bishop SR, Pivik J. The Pain Catastrophizing Scale: Development and validation. *Psychol Assess* 1995;7(4):524-32. doi: 10.1037/1040-3590.7.4.524
52. Bergstrom G, Jensen IB, Bodin L, et al. Reliability and factor structure of the Multidimensional Pain Inventory--Swedish Language Version (MPI-S). *Pain* 1998;75(1):101-10. [published Online First: 1998/04/16]
53. Turk DC, Rudy TE. Toward an empirically derived taxonomy of chronic pain patients: integration of psychological assessment data. *J Consult Clin Psychol* 1988;56(2):233-8. [published Online First: 1988/04/01]
54. Turk DC, Rudy TE. The robustness of an empirically derived taxonomy of chronic pain patients. *Pain* 1990;43(1):27-35. doi: 10.1016/0304-3959(90)90047-h [published Online First: 1990/10/01]
55. McKillop JM, Nielson WR. Improving the usefulness of the Multidimensional Pain Inventory. *Pain Res Manag* 2011;16(4):239-44. doi: 10.1155/2011/873424 [published Online First: 2011/11/08]
56. Gevins A, Cutillo B. Spatiotemporal dynamics of component processes in human working memory. *Electroencephalogr Clin Neurophysiol* 1993;87(3):128-43. doi: 10.1016/0013-4694(93)90119-g [published Online First: 1993/09/01]
57. Fang X, Liu C, Jiang Z. Reference values of gait using APDM movement monitoring inertial sensor system. *R Soc Open Sci* 2018;5(1):170818. doi: 10.1098/rsos.170818 [published Online First: 2018/02/08]
58. Hermens HJ, Frerik B. The SENIAM project (Surface ElectroMyoGraphy for the Non-Invasive Assessment of Muscles) 2020 [cited 2020 November 26]. Available from: http://www.seniam.org/.
59. Hermens HJ, Freriks B, Disselhorst-Klug C, et al. Development of recommendations for SEMG sensors and sensor placement procedures. *Journal of electromyography and kinesiology : official journal of the International Society of Electrophysiological Kinesiology* 2000;10(5):361-74. doi: 10.1016/s1050-6411(00)00027-4 [published Online First: 2000/10/06]
60. Kribus-Shmiel L, Zeilig G, Sokolovski B, et al. How many strides are required for a reliable estimation of temporal gait parameters? Implementation of a new

For peer review only - http://bmjopen.bmj.com/site/about/guidelines.xhtml

algorithm on the phase coordination index. *PLoS One* 2018;13(2):e0192049. doi: 10.1371/journal.pone.0192049 [published Online First: 2018/02/09]

61. Cohen J. Statistical power analysis for the behavioral sciences. 2nd ed. Hillsdale, N.J.: L. Erlbaum Associates 1988.
62. Tzagarakis G, Tsivgoulis S, Papagelopoulos P, et al. Influence of Acute Anterior Cruciate Ligament Deficiency in Gait Variability. *Journal of International Medical Research* 2010;38(2):511-25. doi: 10.1177/147323001003800214
63. Harbourne RT, Stergiou N. Movement variability and the use of nonlinear tools: principles to guide physical therapist practice. *Phys Ther* 2009;89(3):267-82. doi: 10.2522/ptj.20080130 [published Online First: 20090123]
64. Strongman C, Morrison A. A scoping review of non-linear analysis approaches measuring variability in gait due to lower body injury or dysfunction. *Human Movement Science* 2020;69:102562. doi: https://doi.org/10.1016/j.humov.2019.102562
65. Molander P, Dong HJ, Ang B, et al. The role of pain in chronic pain patients' perception of health-related quality of life: a cross-sectional SQRP study of 40,000 patients. *Scand J Pain* 2018;18(3):417-29. doi: 10.1515/sjpain-2018-0003 [published Online First: 2018/05/26]
66. Rice D, Nijs J, Kosek E, et al. Exercise-Induced Hypoalgesia in Pain-Free and Chronic Pain Populations: State of the Art and Future Directions. *The journal of pain : official journal of the American Pain Society* 2019;20(11):1249-66. doi: 10.1016/j.jpain.2019.03.005 [published Online First: 2019/03/25]
67. van Hecke O, Torrance N, Smith BH. Chronic pain epidemiology - where do lifestyle factors fit in? *Br J Pain* 2013;7(4):209-17. doi: 10.1177/2049463713493264 [published Online First: 2013/11/01]

For peer review only

For peer review only - http://bmjopen.bmj.com/site/about/guidelines.xhtml

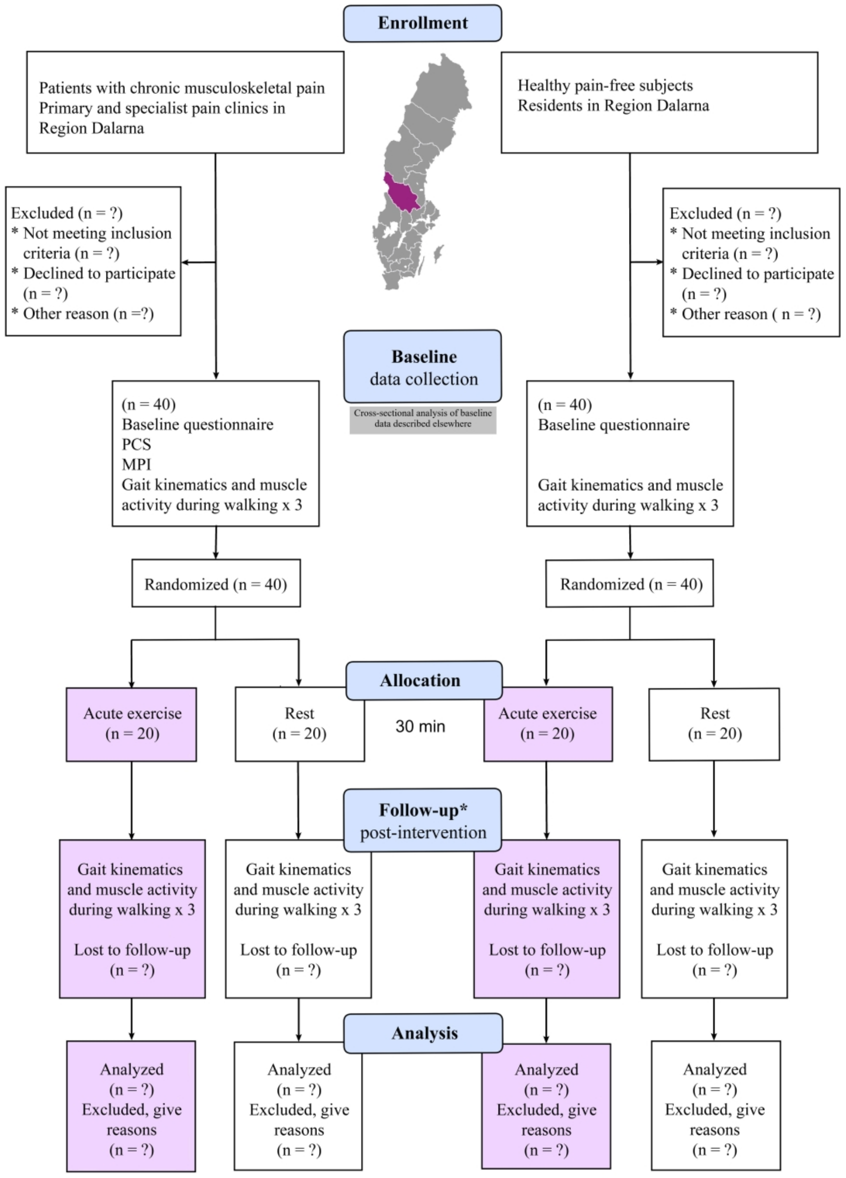

Flowchart

1147x1574mm (96 x 96 DPI)

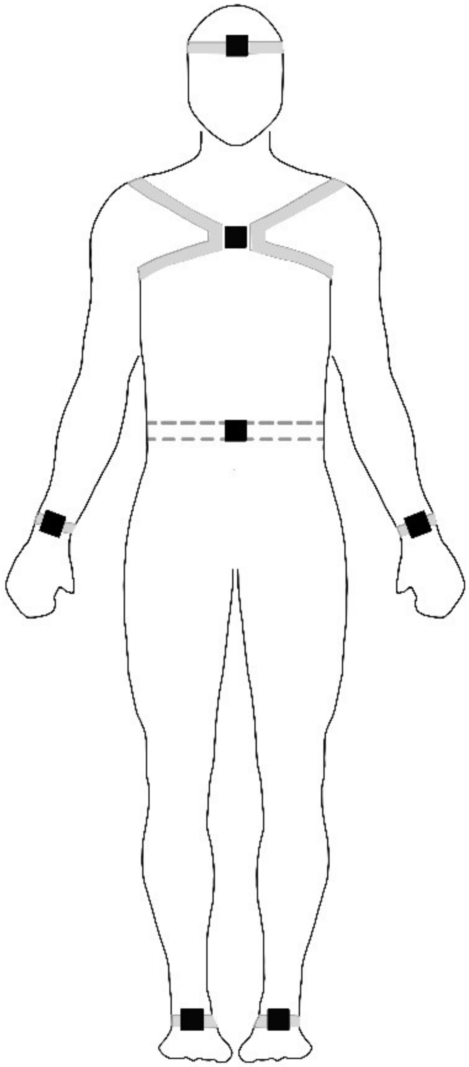

IMU Sensor placement

79x112mm (220 x 220 DPI)

Muscle	EMG electrode locations	Orientation	Illustration
Erector Spinae Neck, bilateral	Placed 20 mm lateral to the median line at the level of C4-C5 spinous process	Vertical	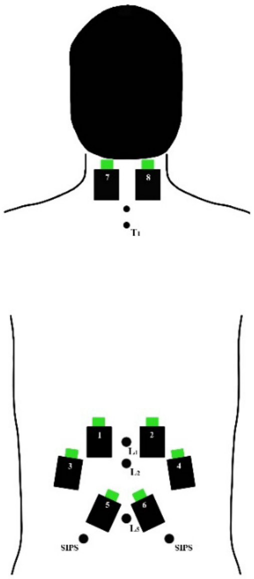
Erector Spinae Longissimus (EL), bilateral	Placed 2 finger widths lateral from the spinous process of L1.	Vertical	
Erector Spinae Iliocostalis (IL), bilateral	Placed 1 finger width medial from a line from the posterior spina iliaca superior to the lowest point of the lower rib, at the level of L2.	Vertical	
Multifidus (MF), bilateral	Placed on and aligned with a line from caudal tip posterior spina iliaca superior to the interspace between L1 and L2 interspace at the level of L5 spinous process (i.e. about 20-30 mm from midline).	Aligned with line, 45° from horizontal	

EMG, electromyography

EMG Sensor placement

390x275mm (79 x 87 DPI)

SPIRIT

STANDARD PROTOCOL ITEMS: RECOMMENDATIONS FOR INTERVENTIONAL TRIALS

Combined SPIRIT 2013 and SPIRIT-PRO 2018 Checklist: Recommended items to address in the clinical trial protocol and related documents by Westergren et al.

Section/item	Item No	Description	Addressed on page number
**Administrative information**			
Title	1	Descriptive title identifying the study design, population, interventions, and, if applicable, trial acronym	1
Trial registration	2a	Trial identifier and registry name. If not yet registered, name of intended registry	2
	2b	All items from the World Health Organization Trial Registration Data Set	2
Protocol version	3	Date and version identifier	2
Funding	4	Sources and types of financial, material, and other support	19
Roles and responsibilities	5a	Names, affiliations, and roles of protocol contributors<br>SPIRIT-PRO<br>Specify the individual(s) responsible for the PRO content of the trial protocol.	1, 19-20
	5b	Name and contact information for the trial sponsor	19 (ej contact info)
	5c	Role of study sponsor and funders, if any, in study design; collection, management, analysis, and interpretation of data; writing of the report; and the decision to submit the report for publication, including whether they will have ultimate authority over any of these activities	19
	5d	Composition, roles, and responsibilities of the coordinating centre, steering committee, endpoint adjudication committee, data management team, and other individuals or groups overseeing the trial, if applicable (see Item 21a for data monitoring committee)	Not applicable
**Introduction**			

For peer review only

Background and rationale	6a	Description of research question and justification for undertaking the trial, including summary of relevant studies (published and unpublished) examining benefits and harms for each intervention	4–6
		SPIRIT-PRO<br>Describe the PRO-specific research question and rationale for PRO assessment and summarize PRO findings in relevant studies	4–6
	6b	Explanation for choice of comparators	5
Objectives	7	Specific objectives or hypotheses<br>SPIRIT-PRO<br>State specific PRO objectives or hypotheses (including relevant PRO concepts/domains).	6 Aims<br>Study hypothesis
Trial design	8	Description of trial design including type of trial (eg, parallel group, crossover, factorial, single group), allocation ratio, and framework (eg, superiority, equivalence, noninferiority, exploratory)	6-7
**Methods: Participants, interventions, and outcomes**			
Study setting	9	Description of study settings (eg, community clinic, academic hospital) and list of countries where data will be collected. Reference to where list of study sites can be obtained	7
Eligibility criteria	10	Inclusion and exclusion criteria for participants. If applicable, eligibility criteria for study centres and individuals who will perform the interventions (eg, surgeons, psychotherapists)	8–9
		SPIRIT-PRO<br>Specify any PRO-specific eligibility criteria (eg, language/reading requirements or prerandomization completion of PRO).<br>If PROs will not be collected from the entire study sample, provide a rationale and describe the method for obtaining the PRO subsample.	9
Interventions	11a	Interventions for each group with sufficient detail to allow replication, including how and when they will be administered	9-14 interventions
	11b	Criteria for discontinuing or modifying allocated interventions for a given trial participant (eg, drug dose change in response to harms, participant request, or improving/worsening disease)	13 (Design) included in ethics approval.
	11c	Strategies to improve adherence to intervention protocols, and any procedures for monitoring adherence (eg, drug tablet return, laboratory tests)	Not Applicable
	11d	Relevant concomitant care and interventions that are permitted or prohibited during the trial	Not Applicable

For peer review only - http://bmjopen.bmj.com/site/about/guidelines.xhtml

Outcomes	12	Primary, secondary, and other outcomes, including the specific measurement variable (eg, systolic blood pressure), analysis metric (eg, change from baseline, final value, time to event), method of aggregation (eg, median, proportion), and time point for each outcome. Explanation of the clinical relevance of chosen efficacy and harm outcomes is strongly recommended	10–13
		SPIRIT-PRO<br>Specify the PRO concepts/domains used to evaluate the intervention (eg, overall health-related quality of life, specific domain, specific symptom) and, for each one, the analysis metric (eg, change from baseline, final value, time to event) and the principal time point or period of interest.	10–13
Participant timeline	13	Time schedule of enrolment, interventions (including any run-ins and washouts), assessments, and visits for participants. A schematic diagram is highly recommended (see Figure)	14-16<br>Figure 1<br>Table 1
		SPIRIT-PRO<br>Include a schedule of PRO assessments, providing a rationale for the time points, and justifying if the initial assessment is not prerandomization. Specify time windows, whether PRO collection is prior to clinical assessments, and, if using multiple questionnaires, whether order of administration will be standardized.	14-16<br>Figure 1<br>Table 1
Sample size	14	Estimated number of participants needed to achieve study objectives and how it was determined, including clinical and statistical assumptions supporting any sample size calculations	7
		SPIRIT-PRO<br>When a PRO is the primary end point, state the required sample size (and how it was determined) and recruitment target (accounting for expected loss to follow-up).<br>If sample size is not established based on the PRO end point, then discuss the power of the principal PRO analyses.	Not Applicable
Recruitment	15	Strategies for achieving adequate participant enrolment to reach target sample size	7

Methods: Assignment of interventions (for controlled trials)

Allocation:

Sequence generation	16a	Method of generating the allocation sequence (eg, computer-generated random numbers), and list of any factors for stratification. To reduce predictability of a random sequence, details of any planned restriction (eg, blocking) should be provided in a separate document that is unavailable to those who enrol participants or assign interventions	8-9
Allocation concealment mechanism	16b	Mechanism of implementing the allocation sequence (eg, central telephone; sequentially numbered, opaque, sealed envelopes), describing any steps to conceal the sequence until interventions are assigned	8-9

Implementation	16c	Who will generate the allocation sequence, who will enrol participants, and who will assign participants to interventions	8-9
Blinding (masking)	17a	Who will be blinded after assignment to interventions (eg, trial participants, care providers, outcome assessors, data analysts), and how	8-9,
	17b	If blinded, circumstances under which unblinding is permissible, and procedure for revealing a participant's allocated intervention during the trial	NA
**Methods: Data collection, management, and analysis**			
Data collection methods	18a	Plans for assessment and collection of outcome, baseline, and other trial data, including any related processes to promote data quality (eg, duplicate measurements, training of assessors) and a description of study instruments (eg, questionnaires, laboratory tests) along with their reliability and validity, if known. Reference to where data collection forms can be found, if not in the protocol	10–13<br>15–17<br>Table 1
		SPIRIT-PRO<br>Justify the PRO instrument to be used and describe domains, number of items, recall period, and instrument scaling and scoring (eg, range and direction of scores indicating a good or poor outcome). Evidence of PRO instrument measurement properties, interpretation guidelines, and patient acceptability and burden should be provided or cited if available, ideally in the population of interest. State whether the measure will be used in accordance with any user manual and specify and justify deviations if planned.	10–13<br>15–17<br>Table 1
		SPIRIT-PRO<br>Include a data collection plan outlining the permitted mode(s) of administration (eg, paper, telephone, electronic, other) and setting (eg, clinic, home, other).	
		SPIRIT-PRO<br>Specify whether more than 1 language version will be used and state whether translated versions have been developed using currently recommended methods	NA
		SPIRIT-PRO<br>When the trial context requires someone other than a trial participant to answer on his or her behalf (a proxy-reported outcome), state and justify the use of a proxy respondent. Provide or cite evidence of the validity of proxy assessment if available.	NA
	18b	Plans to promote participant retention and complete follow-up, including list of any outcome data to be collected for participants who discontinue or deviate from intervention protocols	NA
		SPIRIT-PRO<br>Specify PRO data collection and management strategies for minimizing avoidable missing data.	
		SPIRIT-PRO<br>Describe the process of PRO assessment for participants who discontinue or deviate from the assigned intervention protocol.	

For peer review only - http://bmjopen.bmj.com/site/about/guidelines.xhtml

Data management	19	Plans for data entry, coding, security, and storage, including any related processes to promote data quality (eg, double data entry; range checks for data values). Reference to where details of data management procedures can be found, if not in the protocol	14
Statistical methods	20a	Statistical methods for analysing primary and secondary outcomes. Reference to where other details of the statistical analysis plan can be found, if not in the protocol	14-15
		SPIRIT-PRO<br>State PRO analysis methods, including any plans for addressing multiplicity/type I (α) error.	14-15
	20b	Methods for any additional analyses (eg, subgroup and adjusted analyses)	14-15
	20c	Definition of analysis population relating to protocol non-adherence (eg, as randomised analysis), and any statistical methods to handle missing data (eg, multiple imputation)	14-15
		SPIRIT-PRO<br>State how missing data will be described and outline the methods for handling missing items or entire assessments (eg, approach to imputation and sensitivity analyses).	14-15
**Methods: Monitoring**			
Data monitoring	21a	Composition of data monitoring committee (DMC); summary of its role and reporting structure; statement of whether it is independent from the sponsor and competing interests; and reference to where further details about its charter can be found, if not in the protocol. Alternatively, an explanation of why a DMC is not needed	19
	21b	Description of any interim analyses and stopping guidelines, including who will have access to these interim results and make the final decision to terminate the trial	Not Applicable
Harms	22	Plans for collecting, assessing, reporting, and managing solicited and spontaneously reported adverse events and other unintended effects of trial interventions or trial conduct	13
		SPIRIT-PRO<br>State whether or not PRO data will be monitored during the study to inform the clinical care of individual trial participants and, if so, how this will be managed in a standardized way. Describe how this process will be explained to participants; eg, in the participant information sheet and consent form.	Not applicable

For peer review only - http://bmjopen.bmj.com/site/about/guidelines.xhtml

Auditing	23	Frequency and procedures for auditing trial conduct, if any, and whether the process will be independent from investigators and the sponsor	NA 19
**Ethics and dissemination**			
Research ethics approval	24	Plans for seeking research ethics committee/institutional review board (REC/IRB) approval	2, 19
Protocol amendments	25	Plans for communicating important protocol modifications (eg, changes to eligibility criteria, outcomes, analyses) to relevant parties (eg, investigators, REC/IRBs, trial participants, trial registries, journals, regulators)	14
Consent or assent	26a	Who will obtain informed consent or assent from potential trial participants or authorised surrogates, and how (see Item 32)	11
	26b	Additional consent provisions for collection and use of participant data and biological specimens in ancillary studies, if applicable	NA
Confidentiality	27	How personal information about potential and enrolled participants will be collected, shared, and maintained in order to protect confidentiality before, during, and after the trial	14-15
Declaration of interests	28	Financial and other competing interests for principal investigators for the overall trial and each study site	19
Access to data	29	Statement of who will have access to the final trial dataset, and disclosure of contractual agreements that limit such access for investigators	14-15
Ancillary and post-trial care	30	Provisions, if any, for ancillary and post-trial care, and for compensation to those who suffer harm from trial participation	NA
Dissemination policy	31a	Plans for investigators and sponsor to communicate trial results to participants, healthcare professionals, the public, and other relevant groups (eg, via publication, reporting in results databases, or other data sharing arrangements), including any publication restrictions	19
	31b	Authorship eligibility guidelines and any intended use of professional writers	NA
	31c	Plans, if any, for granting public access to the full protocol, participant-level dataset, and statistical code	20

For peer review only - http://bmjopen.bmj.com/site/about/guidelines.xhtml

Appendices

Informed consent materials	32	Model consent form and other related documentation given to participants and authorised surrogates	11
Biological specimens	33	Plans for collection, laboratory evaluation, and storage of biological specimens for genetic or molecular analysis in the current trial and for future use in ancillary studies, if applicable	Not Applicable

*It is strongly recommended that this checklist be read in conjunction with the SPIRIT 2013 Explanation & Elaboration for important clarification on the items. Amendments to the protocol should be tracked and dated. The SPIRIT checklist is copyrighted by the SPIRIT Group under the Creative Commons "Attribution-NonCommercial-NoDerivs 3.0 Unported" license.

For peer review only

BMJ Open

Acute exercise as active inference in chronic musculoskeletal pain, effects on gait kinematics and muscular activity in patients and healthy participants – A study protocol for a randomised controlled laboratory trial

Journal:	*BMJ Open*
Manuscript ID	bmjopen-2022-069747.R1
Article Type:	Protocol
Date Submitted by the Author:	01-Mar-2023
Complete List of Authors:	Westergren, Jens; Dalarna University, School of Health and Welfare, Department of Sports and Medicine<br>Sjöberg, Veronica; Dalarna University, School of Health and Welfare, Department of Sports and Medicine<br>Vixner, Linda; Hogskolan Dalarna, School of Health and Welfare, Department of Sports and Medicine<br>Nyberg, Roger; Dalarna University, School of Technology and Business Studies<br>Conradsson, David; Karolinska Institutet, Department of Neurobiology, Care Sciences and Society, Division of Physiotherapy; Karolinska Universitetssjukhuset, Medical unit Occupational therapy & Physiotherapy, Theme Women's Health and Allied Health Professional<br>Monnier, Andreas; Dalarna University, School of Health and Welfare, Department of Sports and Medicine; Karolinska Institutet, Department of Neurobiology, Care Sciences and Society, Division of Physiotherapy<br>LoMartire, Riccardo; Uppsala University, Department of Research and Education, Center for Clinical Research Dalarna, Region Dalarna<br>Enthoven, Paul; Linköping University, Department of Health, Medicine and Caring Sciences, Pain and Rehabilitation Centre<br>Äng, Björn; Dalarna University, School of Health and Welfare, Department of Sports and Medicine; Uppsala University, Department of Research and Education, Center for Clinical Research Dalarna, Region Dalarna
**Primary Subject Heading**:	Rehabilitation medicine
Secondary Subject Heading:	Sports and exercise medicine
Keywords:	Back pain < ORTHOPAEDIC & TRAUMA SURGERY, PAIN MANAGEMENT, Musculoskeletal disorders < ORTHOPAEDIC & TRAUMA SURGERY

SCHOLARONE™
Manuscripts

For peer review only - http://bmjopen.bmj.com/site/about/guidelines.xhtml

BMJ Open

Words in text 3995

Words in abstract 286

Tables 1

Figures 3

Appendices 1 (SPIRIT checklist)

Acute exercise as active inference in chronic musculoskeletal pain, effects on gait kinematics and muscular activity in patients and healthy participants – A study protocol for a randomised controlled laboratory trial

Jens Westergren,[1] Veronica Sjöberg,[1] Linda Vixner,[1,] Roger G Nyberg,[1,7] David Moulaee Conradsson,[2,3] Andreas Monnier,[1,2,8] Riccardo LoMartire,[6] Paul Enthoven,[4,5] Björn O Äng.[1,2,6]

[1]Department of Sport and Medicine, School of Health and Welfare, Dalarna University, Falun, Sweden

[2]Department of Neurobiology, Care Sciences and Society, Division of Physiotherapy, Karolinska Institutet, Huddinge, Sweden

[3]Medical unit Occupational therapy & Physiotherapy, Theme Women's Health and Allied Health Professional, Karolinska University Hospital, Stockholm, Sweden

[4]Department of Health, Medicine and Caring Sciences, Physiotherapy, Linköping University, Linköping, Sweden

[5]Pain and Rehabilitation Centre, and Department of Health, Medicine and Caring Sciences, Linköping University, Linköping, Sweden

[6]Department of Research and Education, Center for Clinical Research Dalarna – Uppsala University, Region Dalarna, Falun, Sweden

[7]School of Technology and Business Studies, Dalarna University, Falun, Sweden

[8] Military Academy Karlberg, Swedish Armed Forces, Solna, Sweden

Corresponding author:

For peer review only

29 Jens Westergren
30 Department of Sport and Medicine, School of Health and Welfare, Dalarna University, Falun,
31 Sweden
32 E-Mail: jws@du.se

33

For peer review only

For peer review only - http://bmjopen.bmj.com/site/about/guidelines.xhtml

ABSTRACT

Introduction

Chronic musculoskeletal pain is a highly prevalent, complex and distressing condition that may negatively affect all domains of life. In view of an active inference framework, and resting on the concept of allostasis, human movement per se becomes a prerequisite for health and wellbeing while chronic pain becomes a sign of a system unable to attenuate an allostatic load. Previous studies on different subgroups of chronic pain conditions have demonstrated alterations in gait kinematics and muscle activity, indicating shared disturbances in the motor system from long-term allostatic load. We hypothesize that such alterations exist in heterogenous populations with chronic musculoskeletal pain, and that exposure to acute and controlled exercise, may attenuate these alterations. Therefore, the main aim of this study is to investigate the acute effects of exercise on gait kinematics and activity of the back and neck muscles during diverse walking conditions in patients with chronic musculoskeletal pain compared to a reference sample consisting of healthy participants.

Methods and analysis

This two-sample two-armed parallel randomised controlled laboratory trial will include 40 participants with chronic musculoskeletal pain (> 3 months) and 40 healthy participants. Participants will be randomly allocated to either 30 min of aerobic exercise or rest. Primary outcomes are gait kinematics (walking speed, step frequency, stride length, lumbar rotation, gait stability) and muscular activity (spatial and temporal) of the back and neck during diverse walking conditions. Secondary outcomes are variability of gait kinematics and muscle activity and subjective pain ratings assessed regularly during the trial.

For peer review only - http://bmjopen.bmj.com/site/about/guidelines.xhtml

Ethics and dissemination

The study has been approved by the Regional Ethics Review Board in Uppsala, Sweden (#2018/307/308). Findings will be disseminated via conference presentations, publications in peer-reviewed journals and engagement with patient support groups and clinicians.

Trial Registration: ClinicalTrials.gov; NCT03882333, March 20, 2019.

Keywords:

Chronic Musculoskeletal Pain, Active Inference, Acute Aerobic Exercise, Gait Kinematics, Electromyography

For peer review only

For peer review only - http://bmjopen.bmj.com/site/about/guidelines.xhtml

Strengths and limitations of this study

- To our knowledge, this will be the first randomised controlled laboratory trial investigating the acute effects of aerobic exercise, as active inference, on gait kinematics and muscular activity in a heterogenous group of patients with chronic musculoskeletal pain.
- As long-term aerobic exercise is ultimately the result of regular sessions of aerobic exercise, studying effects induced by a single session of aerobic exercise will provide important insights into exercise and its role in the rehabilitation of chronic pain conditions.
- The study will incorporate objective measures of gait kinematics and muscle activity from continuous overground walking by utilizing modern wireless sensors, thus replicating real world conditions of functional movement.
- While complete blinding is not possible post intervention, due to the nature of exercise, care will be taken to minimize investigator bias by using automated recording protocols.

Based on protocol version 1.0; May 12, 2020.

For peer review only

For peer review only - http://bmjopen.bmj.com/site/about/guidelines.xhtml

INTRODUCTION

Chronic musculoskeletal pain is a highly prevalent and complex condition that continues to challenge clinicians and academics and as such constitutes a major health and wellbeing burden.[1 2] The International Association for the Study of Pain (IASP) defines chronic pain as "pain which has persisted beyond normal tissue healing time", which, in the absence of other factors, is a pain condition lasting for more than three months.[3] Conditions with chronic musculoskeletal pain may be referred to as chronic primary pain[4] and should be seen as a disease in its own right.[5] It can negatively affect physical movement and functioning, sleep, social interaction, quality of life and work ability,[6] in essence all domains of life.

In the light of an active inference framework,[7-9] based on the free energy principle proposed by Friston[10 11] and resting on the concept of allostasis,[12] any living system becomes a model of the world it inhabits. Then, through the process of active inference it attempts to maximize the proof of its own existence (chances of survival) by minimizing prediction errors (variational free energy) through perception and action. The physiological concept of allostasis can be described as achieving stability through change,[12] in which the rate of change influences the process of adaption. In addition, living things may be defined by their ability to move autonomously, be it of bodies, limbs or cells, making movement a key driver of allostasis through sensory palpation of the world. In view of this, human movement, from cell respiration to locomotion, may constitute the major source of active inference upon which the organism builds and maintains its model, making human movement of all sorts pertinent to long-term health and prosperity by facilitating the changes that are needed for stability.

For peer review only - http://bmjopen.bmj.com/site/about/guidelines.xhtml

Building on this notion, in a healthy context acute pain can be viewed as a precision signal for learning and control, with the main function being motivational to direct both short- and long-term motor behaviour away from harm.[13] Chronic musculoskeletal pain on the other hand, is suggestive of a living system in distress and may be explained by the creation and maintenance of abnormal priors that are afforded abnormal attention in a self-evidencing system.[7 14]

This reasoning is – we believe – in line with earlier theories such as the body-self neuromatrix model,[15] the theory of Moving differently in pain,[16] and effectively aligning with concepts such as sensitization[17 18] and ultimately depression[19] within chronic pain populations, as well as recent theory on active interoceptive inference.[20] Then chronic pain can also be viewed as the consequence of an allostatic overload from unresolved prediction errors of the systems lower level predictions about its internal and external milieu.[17 19 21-24] These prediction errors in lower level predictions may manifest themselves at higher levels as fear avoidance behaviour of movement[25] and intolerance of uncertainty,[26] which lead to the further reduction of physical activity.[27-29] This relative sensory deprivation[30 31] that is induced by sedentary behaviour may disable self-organisation normally driven through allostasis, leading to aberrant neuro-signatures (models) of painful states. In essence, chronic musculoskeletal pain becomes a pathophysiology with a self-fulfilling hypothesis of pain as the systems only solution to minimize prediction errors. Building on this notion, physical activity and exercise, or simply human movement, can be viewed as active inference and as such may facilitate a return to allostasis and less painful future states by promoting the flexible reassignment of precision weighting that alters the individual's prediction about their body and their world.[32] This implies that adding physical activity or exercise to a

For peer review only

For peer review only - http://bmjopen.bmj.com/site/about/guidelines.xhtml

rehabilitation program, may simply be reintroducing something essential that was engineered away generations ago.

From this active inference view of chronic pain, it is understandable that a biopsychosocial approach has gained traction in the treatment of chronic pain conditions,[6] [33] [34] [35] since such interdisciplinary treatment delivered by a team of allied health professionals involves a combination of physical activity and exercise, psychological interventions, pharmaceutical treatments, and patient education all acting as active inference[36] enabling a return to less painful states.

Given the fact that the musculoskeletal system is crucial to human locomotion or functional movement, a large body of research has focused on the effects of chronic pain on functional movement. There is now moderate to strong evidence that individuals with persistent low back pain demonstrate differences in gait kinematics,[37] that individuals with non-specific low back pain demonstrate impaired postural control when standing,[38] and that individuals diagnosed with fibromyalgia demonstrate an altered gait compared to healthy individuals.[39] Neuroimaging studies have reported functional brain changes related to sensorimotor impairment in individuals with low back pain,[40] irritable bowel syndrome,[14] fibromyalgia[41] and migraine,[42] suggestive of shared disturbances of the motor system across different diagnoses. The overall finding is that persons with chronic pain conditions walk slower, with a lower step frequency, shorter step-length and with a greater amplitude of back muscle activity.[37 39]

However, based on the notion of a shared disturbance it is currently not known if the altered gait patterns identified in specific sub-groups of chronic pain patients may generalize to wider

For peer review only - http://bmjopen.bmj.com/site/about/guidelines.xhtml

populations of chronic musculoskeletal pain patients and if they could be used as markers of functional movement impairment in these populations. There is a paucity in how exercise, viewed as active inference can have an acute effect on the motor control of walking in humans, especially in adaptions to perturbations such as faster walking speeds or dual tasks and if this is related to long-term sedentary behaviour.

Therefore, the main aim of this study is to investigate the acute effects of exercise as active inference on gait kinematics and activity of the back and neck muscles during diverse walking conditions in patients with chronic musculoskeletal pain compared to a reference sample consisting of participants without chronic musculoskeletal pain.

Our hypothesis is that a wide sample with chronic musculoskeletal pain will walk at lower speeds, lower step frequency and stride lengths but with increased muscle activity in the back and neck and that these differences will increase with task difficulty and begin to attenuate acutely by exercise.

MATERIALS AND METHODS

Trial design

The study consists of a two-sample two-armed parallel randomised controlled laboratory trial (RCT) that will be conducted at Dalarna University, Sweden (Figure 1). The main sample consists of patients with chronic pain who will be randomised into one of two arms: an intervention group performing a single session of acute aerobic exercise (30 min stationary cycling) or a resting control group. The reference sample consists of participants without chronic musculoskeletal pain who will follow a similar RCT procedure, with the purpose being to investigate whether the acute response to exercise is unique for patients with chronic

For peer review only

For peer review only - http://bmjopen.bmj.com/site/about/guidelines.xhtml

pain as our hypothesis proposes, or if it is just a general effect that would be seen any population. The trial will comply with Consolidated Standards of Reporting Trials (CONSORT) and Standard Protocol Items: Recommendations for Interventional Trials guidelines with Patient Reported Outcomes (SPIRIT-PRO).[43-46] A completed SPIRIT-PRO checklist for the trial can be found in the supporting information. Data will be collected between January 2019 and May 2021.

Sample size for this multi project data collection was largely based on studies described elsewhere, in addition a power calculation from right side Erector Spinae Longissimus normalized back muscle activity collected from our first 20 participants confirmed a sample size of n = 40 for a power of 0.80 for $\alpha = 0.05$ (two sided) to identify a 25% difference between patients and participants without musculoskeletal pain (reported elsewhere).

Please insert Figure 1 here

Participants and setting

This trial includes 80 participants. Patients (n=40) that visit primary and specialist pain clinics in Region Dalarna for evaluation or treatment of chronic musculoskeletal pain (> 3 months), as well as an age and gender matched convenience sample of participants without musculoskeletal pain (n=40) will be simultaneously recruited. The physiotherapists treating or evaluating potential participants at the cooperating clinics will act as liaisons to inform patients of the study and those interested in participation will be asked to provide their contact

For peer review only - http://bmjopen.bmj.com/site/about/guidelines.xhtml

204 information. Researchers will then contact all potential participants by telephone and e-mail prior to the laboratory visit, to perform a preliminary screening of eligibility criteria before scheduling a laboratory visit.

Eligibility criteria

Participants aged 18-67 years will be included. Patients will be in assessment and/or treatment of chronic (>3 months) musculoskeletal (neck and/or low back) pain or widespread pain in primary or specialist pain care in Region Dalarna. Exclusion criteria: 1) Any co-existing medical condition that would restrict safely participating in the exercise intervention. 2) Previous spinal surgery, 3) Inability to perform unsupported indoor walking, 4) Inadequate Swedish or/cognitive ability to provide consent and understand information and instructions, 5) Pregnant in second or third trimester or childbirth in the last three months, 6) Allergies to adhesive tape used for sensor application, 7) Major hearing impairment effecting performance of auditory 1-back test, 7) Pain caused by malignancies or systemic diseases, or localized pain in lower extremities only.

Randomisation, allocation concealment and blinding

Participants will be randomly assigned to either the intervention or control group at a 1:1 allocation ratio, using stratified block randomization with a fixed block size of six within the chronic musculoskeletal pain and no chronic pain strata. Allocation information is kept in opaque sealed envelopes and will not be disclosed to participants or researchers until all baseline walk trials are completed. All data collection will be automated in pre-programmed sequences and participant instructions read verbatim to minimize any investigator bias influencing participants behaviour. Participants will not be informed of the hypotheses of the study, and the researcher performing the data analyses will be blinded to group allocation.

For peer review only - http://bmjopen.bmj.com/site/about/guidelines.xhtml

Interventions

The acute aerobic exercise intervention will consist of one 20-minute cycling interval preceded by a five-minute warm-up and followed by a five-minute cool-down (total duration of 30 minutes) and will be performed on a stationary electromagnetically braked cycle ergometer (Schoberer Rad Messtechnik SRM, Jülich, Germany). Self-selected exercise intensity will be guided to moderate intensity by using Borg Ratings of Perceived Exertion (RPE) scale with a target RPE of 13-15 (somewhat hard/ hard).[47] Borg RPE and self-rated pain on VAS (0-100mm) will be recorded every five-minutes during the exercise session. Power output, cadence, and heart rate will be recorded at 2 Hz. Participant will be able to freely regulate cadence and resistance to maintain the target RPE of 13-15, by means of a 14-increment mechanical gear shifter located on the handlebars. The ergometer is equipped with an upright handlebar, flat pedals and a wide padded saddle with an extra gel cover to minimize sitting discomfort.

Participants allocated to the control groups will rest for the equivalent period of 30 minutes in a self-selected comfortable resting position accompanied by staff.

Outcome measures

The primary outcomes (detailed in table 1) are kinematic measures of human movement and muscle activity that will be measured while participants are walking using the following three gait conditions: 1) self-selected habitual gait speed, 2) brisk gait speed, and 3) self-selected

For peer review only

For peer review only - http://bmjopen.bmj.com/site/about/guidelines.xhtml

habitual gait speed with a concurrent cognitive task. Our primary kinematic outcomes are gait speed, step frequency, stride length, lumbar rotation, and gait stability, while primary outcomes for the muscle activity domain will be mean normalized EMG amplitude averaged over aggregated gait cycles.

Our secondary outcome measures are variability of gait kinematics and muscle activity and self-rated current pain intensity using a horizontal 100-mm line Visual Analogue Scale (VAS) with the statement 'no pain at all' at the extreme left, and 'the worst possible pain' at the extreme right (chronic pain sample only).[48 49]

Table 1. Outcomes and assessments

			Baseline	Intervention	Follow-up
Baseline measures	Variable or assessment	Data Source			
Participant characteristics	Age, sex, body mass index (weight, height), educational level, work status, physical activity.	SAQ+ PT	X		
Pain catastrophizing †	Pain Catastrophizing Scale (PCS)	SAQ	X		
Effects on everyday life †	Multidimensional Pain Inventory (MPI)	SAQ	X		
Primary outcome measures					
**Gait kinematics**<br>- Gait speed (m/s)<br>- Step frequency (steps min^{-1})<br>- Stride length (cm)<br>- Lumbar rotation (°)<br>- Lateral step variability (three consecutive steps, the perpendicular deviation of the middle foot placement from the line connecting the first and the third step)<br><br>**Back and neck muscle activity**<br>- Normalized mean muscle activity of all completed gait cycles per trial<br>- Back and neck normalized muscle activity at each 10 percentile of gait cycle	Gait kinematics with IMU-based sensors and wireless surface EMG, aggregated from minimum 50 gait cycles<br>-Self -selected habitual gait speed<br>- Brisk gait speed‡<br>- Self-selected habitual gait speed with concurrent cognitive task (1-back test) ‡	PT	X		X
Secondary outcome measures					
**Variability of gait kinematics**<br>- Gait speed (m/s)<br>- Step frequency (steps min^{-1})<br>- Stride length (cm)<br>- Lumbar rotation (°)<br>- Lateral step variability<br><br>**Variability of muscle activity**<br>- Normalized mean per trial	Non-linear analysis of gait kinematics with IMU-based sensors and wireless surface EMG, aggregated from minimum 50 gait cycles<br>-Self -selected habitual gait speed<br>- Brisk gait speed‡<br>- Self-selected habitual gait speed with concurrent cognitive task (1-back test) ‡	PT	X		X

For peer review only

For peer review only - http://bmjopen.bmj.com/site/about/guidelines.xhtml

- Balance of left/right at each 10 percentile of gait cycle<br>- Back and neck normalized during stance and swing phase<br>- Number of muscle activity spikes (>100% normalized per complete gait cycle)					
- Pain intensity†	VAS (0-100)	SAQ	X	X	X

Data source: Self-administered questionnaire (SAQ), physical testing (PT).

VAS, Visual Analogue Scale; administered after every gait test. RPE, Rating of Perceived Exertion; every 5 minutes during the intervention; IMU, Inertial Monitoring Unit; EMG, Electromyography.

† Only measured in patients with chronic musculoskeletal pain

‡ Brisk gait speed and self-selected gait speed with the 1-back test will be performed in random order

For peer review only

For peer review only - http://bmjopen.bmj.com/site/about/guidelines.xhtml

Trial procedure, instruments and data collection

The entire trial procedure at the laboratory is expected to take approximately three hours and will be supervised by two researchers. Upon arrival at the laboratory, eligibility screening, presentation of the study procedures and collection of informed consent will be performed. Participants will complete the following questionnaires before commencing any testing: 1) Participant characteristics (age, sex, educational level, occupational status, pain locations, pain duration) including two validated items with questions about physical activity.[50] Participants with chronic musculoskeletal pain will also complete the following two questionnaires relating to pain experiences: 2) The Pain Catastrophizing Scale (PCS)[51] which contains 13 items regarding patients thoughts and feelings associated with pain, with a total score ranging from 0-52, with higher scores representing higher pain catastrophizing and 3) The West Haven-Yale Multidimensional Pain Inventory (MPI), which is a questionnaire that measures the psychosocial, cognitive, and behavioural effects of chronic pain.[52-55]

A researcher will then record participant body height using a stadiometer (Harpenden Stadiometer, Holtain Limited, Crosswell, UK) and body weight using a weighing scale (Midrics, Sartorius AG, Goettingen, Germany). Participants will be introduced to the 1-back test[56] and listen to a 30-second practice sequence, then two 30-second 1-back baseline tests will be recorded with participants in a seated position. The 1-back test will be performed with a pair of minimally intrusive wireless handsfree on-ear headphones connected via Bluetooth to a PC using Audacity Software v. 2.3.0. Participants will listen to an audio file consisting of a randomized number sequence and will be instructed to respond as quickly and accurately as possible to audio prompts, repeating the digit before the last one in the consecutive sequence during the entire 1-back walking trial. All responses will be recorded.

For peer review only - http://bmjopen.bmj.com/site/about/guidelines.xhtml

After this, seven wireless inertial monitoring unit (IMU) sensors (OPAL, APDM INC, OR, USA) incorporating 3-axis- gyroscope, accelerometer and magnetometer will be applied on participants limbs using elastic straps as specified in figure 2. Gait kinematics will be streamed and logged wirelessly from each sensor at 128Hz during all trials and will be automatically processed via Moveo Explorer ™ software to extract gait variables specified as primary or secondary outcomes according to table 1.[57] Participants will be given standardized instructions prior to starting each gait trial in order to comply with the initial calibration procedure described by the manufacturer.

Please insert Figure 2 here

Wireless electromyography (EMG) sensors (Delsys Trigno, Delsys Inc., Boston, MA, USA) will be positioned according to anatomical landmarks detailed in figure 3, as per SENIAM guidelines [58 59] and data recorded at 1111 Hz. Skin will be shaved and cleansed with a chlorhexidine digluconate ethanol cutaneous solution (Klorhexidinsprit 5mg/ml, Fresenius Kabi AB, Uppsala, Sweden). To obtain normalizing (reference) muscle activity, each participant will perform four standardized submaximal standing trunk flexion (hip flexion to 45° from neutral standing position) muscle contraction tests, interspersed by a 60-second rest. EMG sensors will be removed after baseline measurements and reapplied prior to the follow-up walking trials. Before removal sensor positions will be marked with a marker pen to facilitate accurate reapplication.

Please insert Figure 3 here

For peer review only - http://bmjopen.bmj.com/site/about/guidelines.xhtml

All gait trials will be performed indoors in a continuous fashion, on a 15-meter standardised walkway with tape markings on the floor indicating starting and 180° turning points. Trials will have a minimum duration of two minutes and finish when participants cross the start line with a time exceeding two minutes. Participants will initially perform a familiarization trial at a self-selected habitual speed followed by a short rest if needed, to avoid any accumulated pain or fatigue. After this, participants will proceed to walk the 15-meter walkway under the three different conditions described above: 1) an initial self-selected habitual gait speed, 2) a brisk gait speed and 3) a self-selected habitual gait speed with a concurrent cognitive task (1-back test). The order of performance of the brisk and 1-back walking conditions (2 or 3) will be randomised and the allocation will be kept concealed to the participants until the initial condition with self-selected habitual gait speed has been performed. For the brisk walking condition, participants will be instructed to walk faster than they habitually do, as they imagine themselves as slightly late for a bus they want to catch. The self-rated pain intensity (VAS) of participants with chronic musculoskeletal pain will be recorded after each walk trial. The risk of complication due to measurements or interventions is considered to be low, but any adverse or serious adverse events will be logged and reported.

For peer review only

Data management

Participant questionaries will be checked for completeness and congruity before data is entered into the database. Gait kinematics will be aggregated for all valid gait cycles of each gait condition. For each individual, a minimum of 50 gait cycles will be used and aggregated prior to analysis.[60] Back and neck muscle activity (root-mean square, RMS) synchronized to gait cycles and aggregated from each gait condition will be normalized as percentage RMS against reference contractions.

For peer review only - http://bmjopen.bmj.com/site/about/guidelines.xhtml

Statistical analysis

Participant characteristics will be reported using descriptive statistics. The statistical analyses will be performed on an intention to treat basis and potential differences between the intervention groups and the control groups. Analysis will primarily be conducted with variance analysis with respect to both data quality and distribution (data assumptions), and with the addition of repeated measurements where applicable. Log transformations of data will be made as appropriate. Data will be presented with means and variances. To explore the magnitude of the effect of interventions, effect sizes will be calculated.[61] Lastly, non-linear analysis using Gait Evaluation Differential Entropy Method (GEDEM)[62] will be used to assess variability of gait kinematic and muscular activity.[62-64]

Ethics and dissemination

The study has been approved by the Regional Ethics Review Board in Uppsala, Sweden (#2018/307/308). Findings will be disseminated via conference presentations, publications in peer-reviewed journals and engagement with patient support groups and clinicians.

Patient and public involvement

The burden of the study procedures has been assessed by patients during preliminary pilot work, primarily to assess the following areas: the feasibility of the cycling exercise intervention, the baseline questionnaires, and the instrumentation to capture gait kinematics and EMG-data. Study participants will be invited to a briefing where the results of the study will be presented. In addition, study participants will be given a summary of the results written in Swedish. Results will be presented at the annual meeting of the Swedish National Registry for Chronic Pain Rehabilitation where patient groups are represented.

For peer review only - http://bmjopen.bmj.com/site/about/guidelines.xhtml

DISCUSSION

The current study is to our knowledge the first laboratory RCT to investigate the acute effect of aerobic exercise on gait kinematics and muscle activity in participants with chronic musculoskeletal pain compared to reference participants from a perspective of exercise as active inference. Our two-sample two-armed parallel design allows for a cross comparison of effects in patients and healthy participants that will follow the same study protocol and will contribute to our understanding of how chronic musculoskeletal pain manifest on measures of human motor behaviour and how exercise can influence this in a rehabilitation setting. Knowledge of how gait kinematics and back and neck muscular activity may change during different gait conditions and from aerobic exercise may contribute to the understanding of how exercise therapy influences our generative models in the process attenuates allostatic load through active inference. The use of modern, miniaturized, and completely wireless sensors during overground walking replicates real world conditions, hence strengthening the ecological validity of our results. Objective measurements of movement and muscular activity during diverse walking conditions have been chosen as primary outcomes since such a methodology can both uniquely and sensitively show and quantify deviating activation patterns as validly reflected in every day functional movements and commonly performed physical activities.

Participants

Selection bias is difficult to completely overcome in exercise intervention studies as volunteering participants will likely be positively biased towards exercise in general and motivation to participate in an exercise intervention is also likely influenced by the severity of

For peer review only - http://bmjopen.bmj.com/site/about/guidelines.xhtml

symptoms experienced when asked to volunteer for the study. We will compare and validate our sample baseline characteristics with a new nationwide large database including 60,000 patients with chronic pain.[65]

Concerning our reference participants without chronic musculoskeletal pain, we aim to create a sample that is sex and age matched with our chronic musculoskeletal pain sample. It will serve as a reference sample for all our outcomes – can our hypothesis on the acute effects of exercise in our sample with chronic pain be verified or may unexpected results emerge when healthy participants are subjected to the same intervention?

Intervention

Viewing this exercise intervention as active inference will hopefully add knowledge on how acute movement through exercise may promote an individual's predictions about their body and their world by creating a platform for belief updating. Our use of a cycling modality with a rather different muscle recruitment and joint loading pattern compared to walking may be beneficial, especially since stationary cycling may be more palatable to participants than running at the same intensity as it may lead to an undesired level of fatigue, fear, or increased pain.

In addition, the aerobic exercise intervention in the current study reflects recommendations in exercise guidelines and allows an easy progression of rehabilitation in terms of both intensity and duration.[66] Since a bicycle can be used for therapy, recreation, and transportation, there are opportunities for health care professionals to facilitate changes in lifestyle that have the potential to mitigate morbidity and function in patients with chronic pain,[67] simultaneously reducing the risks and impact of common co-morbidities.

For peer review only

For peer review only - http://bmjopen.bmj.com/site/about/guidelines.xhtml

Outcomes

With the foundation of this project being objective measures of overground walking, it adheres well to the Initiative on Methods, Measurement and Pain Assessment in Clinical Trials (IMMPACT) which recommends that all chronic-pain trials should include measures of physical functioning.[49] This project is expected to result in increased knowledge of how acute exercise effects subsequent functional tasks, here measured during different walking conditions.

Potential impact

Our proposed project will deepen our understanding on the topic of alterations in functional movement in chronic musculoskeletal pain conditions.

Perspective

Results from the current study may help us reach a better understanding how acute aerobic exercise acts as active inference to attenuate chronic pain by facilitating allostasis. This knowledge will help us formulate better questions as to how generative models in chronic pain conditions are formed, maintained, and updated through movement, or lack thereof.

For peer review only - http://bmjopen.bmj.com/site/about/guidelines.xhtml

Author Contributions

All authors meet the ICMJE criteria for authorship. JW, VS, BÄ and LV take responsibility for the integrity of the study. All authors were involved in the study´s concept and design. JW and VS will be involved in acquisition of data. JW, PE, AM, RLM, DMC, RGN and BÄ were involved in drafting and revising the article critically for important intellectual content. All authors were involved in manuscript revision and read and approved the final version to be published.

Funding

This work is supported by the Swedish Research Council for Health (2015-02512), Working life and Welfare (FORTE; 2017-00177), and Dalarna University (N/A), Falun, Sweden. The funders had no role in study design and will have no role in any part of the study or reporting of the results.

Competing interests

None declared.

Patient consent for publication

Not required.

Ethics approval

The study has been approved by the Regional Ethics Review Board in Uppsala, Sweden; approval ID 2018/307/308.

For peer review only

For peer review only - http://bmjopen.bmj.com/site/about/guidelines.xhtml

Data sharing statement

Not applicable

Open access

This is an open access article distributed in accordance with the Creative Commons Attribution Non-Commercial (CC BY-NC 4.0) license, which permits others to distribute, remix, adapt, build upon this work non-commercially, and license their derivative works on different terms, provided the original work is properly cited, appropriate credit is given, any changes made indicated, and the use is non-commercial.

See: http://creativecommons.org/licenses/by-nc/4.0/.

FIGURE CAPTIONS:

Fig. 1. Participant flowchart.
PCS, Pain Catastrophizing Scale; MPI, Multidimensional Pain Inventory.

Figure 2. IMU-sensor placement on the forehead, sternum, lumbar spine, wrists, and feet. IMU, Inertial monitoring unit. Adapted with permission by APDM, INC.

Figure 3. Overview wireless electrode placement for surface EMG recording of the trunk and neck musculature according to SENIAM guidelines (http://www.seniam.org/)

For peer review only - http://bmjopen.bmj.com/site/about/guidelines.xhtml

For peer review only

REFERENCES

1. Leadley RM, Armstrong N, Lee YC, et al. Chronic diseases in the European Union: the prevalence and health cost implications of chronic pain. *Journal of pain & palliative care pharmacotherapy* 2012;26(4):310-25. doi: 10.3109/15360288.2012.736933 [published Online First: 2012/12/12]
2. Global Burden of Disease Study Collaborators. Global, regional, and national incidence, prevalence, and years lived with disability for 328 diseases and injuries for 195 countries, 1990-2016: a systematic analysis for the Global Burden of Disease Study 2016. *Lancet* 2017;390(10100):1211-59. doi: 10.1016/S0140-6736(17)32154-2 [published Online First: 2017/09/19]
3. Raja SN, Carr DB, Cohen M, et al. The revised International Association for the Study of Pain definition of pain: concepts, challenges, and compromises. *Pain* 2020;161(9):1976-82. doi: 10.1097/j.pain.0000000000001939 [published Online First: 2020/07/23]
4. Nicholas M, Vlaeyen JWS, Rief W, et al. The IASP classification of chronic pain for ICD-11: chronic primary pain. *Pain* 2019;160(1):28-37. doi: 10.1097/j.pain.0000000000001390 [published Online First: 2018/12/27]
5. Niv D, Devor M. Chronic pain as a disease in its own right. *Pain Pract* 2004;4(3):179-81. doi: 10.1111/j.1533-2500.2004.04301.x
6. Ahlberg M. Rehabilitering vid långvarig smärta: En systematisk litteraturöversikt (SBU; Swedish agency for health technology assessment and assessment of social services). 2010
7. De Ridder D, Adhia D, Vanneste S. The anatomy of pain and suffering in the brain and its clinical implications. *Neuroscience & Biobehavioral Reviews* 2021;130:125-46. doi: https://doi.org/10.1016/j.neubiorev.2021.08.013
8. Ongaro G, Kaptchuk TJ. Symptom perception, placebo effects, and the Bayesian brain. *Pain* 2019;160(1):1-4. doi: 10.1097/j.pain.0000000000001367
9. Pezzulo G, Parr T, Friston K. The evolution of brain architectures for predictive coding and active inference. *Philos Trans R Soc Lond B Biol Sci* 2022;377(1844):20200531. doi: 10.1098/rstb.2020.0531 [published Online First: 20211227]
10. Friston K. The history of the future of the Bayesian brain. *Neuroimage* 2012;62(2):1230-3. doi: 10.1016/j.neuroimage.2011.10.004 [published Online First: 2011/10/26]
11. Friston K. Life as we know it. *J R Soc Interface* 2013;10(86):20130475. doi: 10.1098/rsif.2013.0475 [published Online First: 20130703]
12. Sterling P, Eyer J. Handbook of Life Stress, Cognition and Health Edited by S. Fisher and J. Reason Copyright 1988 John Wiley 8. Sons, 1988.
13. Seymour B. Pain: A Precision Signal for Reinforcement Learning and Control. *Neuron* 2019;101(6):1029-41. doi: 10.1016/j.neuron.2019.01.055
14. Nisticò V, Rossi RE, D'Arrigo AM, et al. Functional neuroimaging in Irritable Bowel Syndrome: a systematic review highlights common brain alterations with Functional Movement Disorders. *J Neurogastroenterol Motil* 2022 doi: 10.5056/jnm21079 [published Online First: 20220222]
15. Melzack R, Katz J. Pain. *Wiley Interdisciplinary Reviews: Cognitive Science* 2013;4(1):1-15. doi: 10.1002/wcs.1201

For peer review only

For peer review only - http://bmjopen.bmj.com/site/about/guidelines.xhtml

16. Hodges PW, Tucker K. Moving differently in pain: a new theory to explain the adaptation to pain. *Pain* 2011;152(3 Suppl):S90-8. doi: 10.1016/j.pain.2010.10.020 [published Online First: 2010/11/23]
17. Wallden M, Nijs J. Before & beyond the pain – Allostatic load, central sensitivity and their role in health and function. *Journal of Bodywork and Movement Therapies* 2021;27:388-92. doi: https://doi.org/10.1016/j.jbmt.2021.04.003
18. Wallden M, Nijs J. Applying the understanding of central sensitization in practice. *Journal of Bodywork and Movement Therapies* 2021;27:723-30. doi: 10.1016/j.jbmt.2021.04.004
19. Arnaldo I, Corcoran AW, Friston KJ, et al. Stress and its sequelae: An active inference account of the etiological pathway from allostatic overload to depression. *Neuroscience and biobehavioral reviews* 2022;135:104590. doi: 10.1016/j.neubiorev.2022.104590 [published Online First: 20220218]
20. Fermin ASR, Friston K, Yamawaki S. An insula hierarchical network architecture for active interoceptive inference. *R Soc Open Sci* 2022;9(6):220226. doi: 10.1098/rsos.220226 [published Online First: 20220629]
21. Craig AD. How do you feel? Interoception: the sense of the physiological condition of the body. *Nature Reviews Neuroscience* 2002;3(8):655-66. doi: 10.1038/nrn894
22. Craig AD. A new view of pain as a homeostatic emotion. *Trends Neurosci* 2003;26(6):303-7. doi: 10.1016/s0166-2236(03)00123-1 [published Online First: 2003/06/12]
23. Wallden M, Sisson M. Modern disintegration and primal connectivity. *Journal of Bodywork and Movement Therapies* 2019;23(2):359-65. doi: 10.1016/j.jbmt.2019.03.003
24. McEwen BS. Protective and damaging effects of stress mediators: central role of the brain. *Dialogues Clin Neurosci* 2006;8(4):367-81. doi: 10.31887/DCNS.2006.8.4/bmcewen
25. Ellingsen DM, Napadow V, Protsenko E, et al. Brain Mechanisms of Anticipated Painful Movements and Their Modulation by Manual Therapy in Chronic Low Back Pain. *J Pain* 2018;19(11):1352-65. doi: 10.1016/j.jpain.2018.05.012 [published Online First: 20180703]
26. López-Martínez AE, Ramírez-Maestre C, Serrano-Ibáñez ER, et al. Intolerance of uncertainty moderates the relationship between catastrophizing, anxiety, and perceived pain in people with chronic nononcological pain. *Pain Med* 2022 doi: 10.1093/pm/pnac030 [published Online First: 20220216]
27. Parker R, Bergman E, Mntambo A, et al. Levels of physical activity in people with chronic pain. *S Afr J Physiother* 2017;73(1):323. doi: 10.4102/sajp.v73i1.323 [published Online First: 20170331]
28. McBeth J, Nicholl BI, Cordingley L, et al. Chronic widespread pain predicts physical inactivity: results from the prospective EPIFUND study. *European journal of pain (London, England)* 2010;14(9):972-9. doi: 10.1016/j.ejpain.2010.03.005 [published Online First: 2010/04/20]
29. Jones J, Rutledge DN, Jones KD, et al. Self-assessed physical function levels of women with fibromyalgia: a national survey. *Womens Health Issues* 2008;18(5):406-12. doi: 10.1016/j.whi.2008.04.005 [published Online First: 20080823]
30. Blitz B, Lowenthal M. The role of sensory restriction in problems with chronic pain. *J Chronic Dis* 1966;19(11):1119-25.

For peer review only - http://bmjopen.bmj.com/site/about/guidelines.xhtml

31. Mohan A, Vanneste S. Adaptive and maladaptive neural compensatory consequences of sensory deprivation-From a phantom percept perspective. *Prog Neurobiol* 2017;153:1-17. doi: 10.1016/j.pneurobio.2017.03.010 [published Online First: 20170411]
32. Tabor A, Burr C. Bayesian Learning Models of Pain: A Call to Action. *Current Opinion in Behavioral Sciences* 2019;26:54-61. doi: https://doi.org/10.1016/j.cobeha.2018.10.006
33. Kim J, Esteves JE, Cerritelli F, et al. An Active Inference Account of Touch and Verbal Communication in Therapy. *Front Psychol* 2022;13:828952. doi: 10.3389/fpsyg.2022.828952 [published Online First: 20220520]
34. Nicholas M. Task Force on Multimodal Pain Treatment Defines Terms for Chronic Pain Care. cited 2018-02-13. 2017
35. Kamper SJ, Apeldoorn AT, Chiarotto A, et al. Multidisciplinary biopsychosocial rehabilitation for chronic low back pain: Cochrane systematic review and meta-analysis. *BMJ* 2015;350:h444. doi: 10.1136/bmj.h444
36. McParlin Z, Cerritelli F, Friston KJ, et al. Therapeutic Alliance as Active Inference: The Role of Therapeutic Touch and Synchrony. *Frontiers in Psychology* 2022;13 doi: 10.3389/fpsyg.2022.783694
37. Smith JA, Stabbert H, Bagwell JJ, et al. Do people with low back pain walk differently? A systematic review and meta-analysis. *J Sport Health Sci* 2022 doi: 10.1016/j.jshs.2022.02.001 [published Online First: 20220210]
38. Koch C, Hänsel F. Non-specific Low Back Pain and Postural Control During Quiet Standing-A Systematic Review. *Front Psychol* 2019;10:586. doi: 10.3389/fpsyg.2019.00586 [published Online First: 20190322]
39. Carrasco-Vega E, Ruiz-Muñoz M, Cuesta-Vargas A, et al. Individuals with fibromyalgia have a different gait pattern and a reduced walk functional capacity: a systematic review with meta-analysis. *PeerJ* 2022;10:e12908. doi: 10.7717/peerj.12908 [published Online First: 20220321]
40. Goossens N, Rummens S, Janssens L, et al. Association Between Sensorimotor Impairments and Functional Brain Changes in Patients With Low Back Pain: A Critical Review. *Am J Phys Med Rehabil* 2018;97(3):200-11. doi: 10.1097/phm.0000000000000859 [published Online First: 2017/11/08]
41. Cheng JC, Anzolin A, Berry M, et al. Dynamic Functional Brain Connectivity Underlying Temporal Summation of Pain in Fibromyalgia. *Arthritis Rheumatol* 2022;74(4):700-10. doi: 10.1002/art.42013 [published Online First: 20220310]
42. Karsan N, Goadsby PJ. Migraine Is More Than Just Headache: Is the Link to Chronic Fatigue and Mood Disorders Simply Due to Shared Biological Systems? *Front Hum Neurosci* 2021;15:646692. doi: 10.3389/fnhum.2021.646692 [published Online First: 20210603]
43. Calvert M, Kyte D, Mercieca-Bebber R, et al. Guidelines for Inclusion of Patient-Reported Outcomes in Clinical Trial Protocols: The SPIRIT-PRO Extension. *JAMA* 2018;319(5):483-94. doi: 10.1001/jama.2017.21903
44. Chan AW, Tetzlaff JM, Altman DG, et al. SPIRIT 2013 statement: defining standard protocol items for clinical trials. *Ann Intern Med* 2013;158(3):200-7. doi: 10.7326/0003-4819-158-3-201302050-00583 [published Online First: 2013/01/09]

For peer review only - http://bmjopen.bmj.com/site/about/guidelines.xhtml

45. Schulz KF, Altman DG, Moher D. CONSORT 2010 statement: updated guidelines for reporting parallel group randomised trials. *BMJ (Clinical research ed)* 2010;340:c332. doi: 10.1136/bmj.c332 [published Online First: 2010/03/25]

46. Gamble C, Krishan A, Stocken D, et al. Guidelines for the Content of Statistical Analysis Plans in Clinical Trials. *JAMA* 2017;318(23):2337-43. doi: 10.1001/jama.2017.18556 [published Online First: 2017/12/21]

47. Borg G. Perceived exertion as an indicator of somatic stress. *Scand J Rehabil Med* 1970;2(2):92-8.

48. Huskisson EC. Measurement of pain. *Lancet* 1974;2:1127-31.

49. Taylor AM, Phillips K, Patel KV, et al. Assessment of physical function and participation in chronic pain clinical trials: IMMPACT/OMERACT recommendations. *Pain* 2016;157(9):1836-50. doi: 10.1097/j.pain.0000000000000577 [published Online First: 2016/04/09]

50. Brumagne S, Janssens L, Knapen S, et al. Persons with recurrent low back pain exhibit a rigid postural control strategy. *European spine journal : official publication of the European Spine Society, the European Spinal Deformity Society, and the European Section of the Cervical Spine Research Society* 2008;17(9):1177-84. doi: 10.1007/s00586-008-0709-7 [published Online First: 20080702]

51. Sullivan MJL, Bishop SR, Pivik J. The Pain Catastrophizing Scale: Development and validation. *Psychol Assess* 1995;7(4):524-32. doi: 10.1037/1040-3590.7.4.524

52. Bergstrom G, Jensen IB, Bodin L, et al. Reliability and factor structure of the Multidimensional Pain Inventory--Swedish Language Version (MPI-S). *Pain* 1998;75(1):101-10. [published Online First: 1998/04/16]

53. Turk DC, Rudy TE. Toward an empirically derived taxonomy of chronic pain patients: integration of psychological assessment data. *J Consult Clin Psychol* 1988;56(2):233-8. [published Online First: 1988/04/01]

54. Turk DC, Rudy TE. The robustness of an empirically derived taxonomy of chronic pain patients. *Pain* 1990;43(1):27-35. doi: 10.1016/0304-3959(90)90047-h [published Online First: 1990/10/01]

55. McKillop JM, Nielson WR. Improving the usefulness of the Multidimensional Pain Inventory. *Pain Res Manag* 2011;16(4):239-44. doi: 10.1155/2011/873424 [published Online First: 2011/11/08]

56. Gevins A, Cutillo B. Spatiotemporal dynamics of component processes in human working memory. *Electroencephalogr Clin Neurophysiol* 1993;87(3):128-43. doi: 10.1016/0013-4694(93)90119-g [published Online First: 1993/09/01]

57. Fang X, Liu C, Jiang Z. Reference values of gait using APDM movement monitoring inertial sensor system. *R Soc Open Sci* 2018;5(1):170818. doi: 10.1098/rsos.170818 [published Online First: 2018/02/08]

58. Hermens HJ, Frerik B. The SENIAM project (Surface ElectroMyoGraphy for the Non-Invasive Assessment of Muscles) 2020 [cited 2020 November 26]. Available from: http://www.seniam.org/.

59. Hermens HJ, Freriks B, Disselhorst-Klug C, et al. Development of recommendations for SEMG sensors and sensor placement procedures. *Journal of electromyography and kinesiology : official journal of the International Society of Electrophysiological Kinesiology* 2000;10(5):361-74. doi: 10.1016/s1050-6411(00)00027-4 [published Online First: 2000/10/06]

60. Kribus-Shmiel L, Zeilig G, Sokolovski B, et al. How many strides are required for a reliable estimation of temporal gait parameters? Implementation of a new

For peer review only - http://bmjopen.bmj.com/site/about/guidelines.xhtml

algorithm on the phase coordination index. *PLoS One* 2018;13(2):e0192049. doi: 10.1371/journal.pone.0192049 [published Online First: 2018/02/09]

61. Cohen J. Statistical power analysis for the behavioral sciences. 2nd ed. Hillsdale, N.J.: L. Erlbaum Associates 1988.
62. Tzagarakis G, Tsivgoulis S, Papagelopoulos P, et al. Influence of Acute Anterior Cruciate Ligament Deficiency in Gait Variability. *Journal of International Medical Research* 2010;38(2):511-25. doi: 10.1177/147323001003800214
63. Harbourne RT, Stergiou N. Movement variability and the use of nonlinear tools: principles to guide physical therapist practice. *Phys Ther* 2009;89(3):267-82. doi: 10.2522/ptj.20080130 [published Online First: 20090123]
64. Strongman C, Morrison A. A scoping review of non-linear analysis approaches measuring variability in gait due to lower body injury or dysfunction. *Human Movement Science* 2020;69:102562. doi: https://doi.org/10.1016/j.humov.2019.102562
65. Molander P, Dong HJ, Ang B, et al. The role of pain in chronic pain patients' perception of health-related quality of life: a cross-sectional SQRP study of 40,000 patients. *Scand J Pain* 2018;18(3):417-29. doi: 10.1515/sjpain-2018-0003 [published Online First: 2018/05/26]
66. Rice D, Nijs J, Kosek E, et al. Exercise-Induced Hypoalgesia in Pain-Free and Chronic Pain Populations: State of the Art and Future Directions. *J Pain* 2019;20(11):1249-66. doi: 10.1016/j.jpain.2019.03.005 [published Online First: 20190321]
67. van Hecke O, Torrance N, Smith BH. Chronic pain epidemiology - where do lifestyle factors fit in? *Br J Pain* 2013;7(4):209-17. doi: 10.1177/2049463713493264 [published Online First: 2013/11/01]

For peer review only

For peer review only - http://bmjopen.bmj.com/site/about/guidelines.xhtml

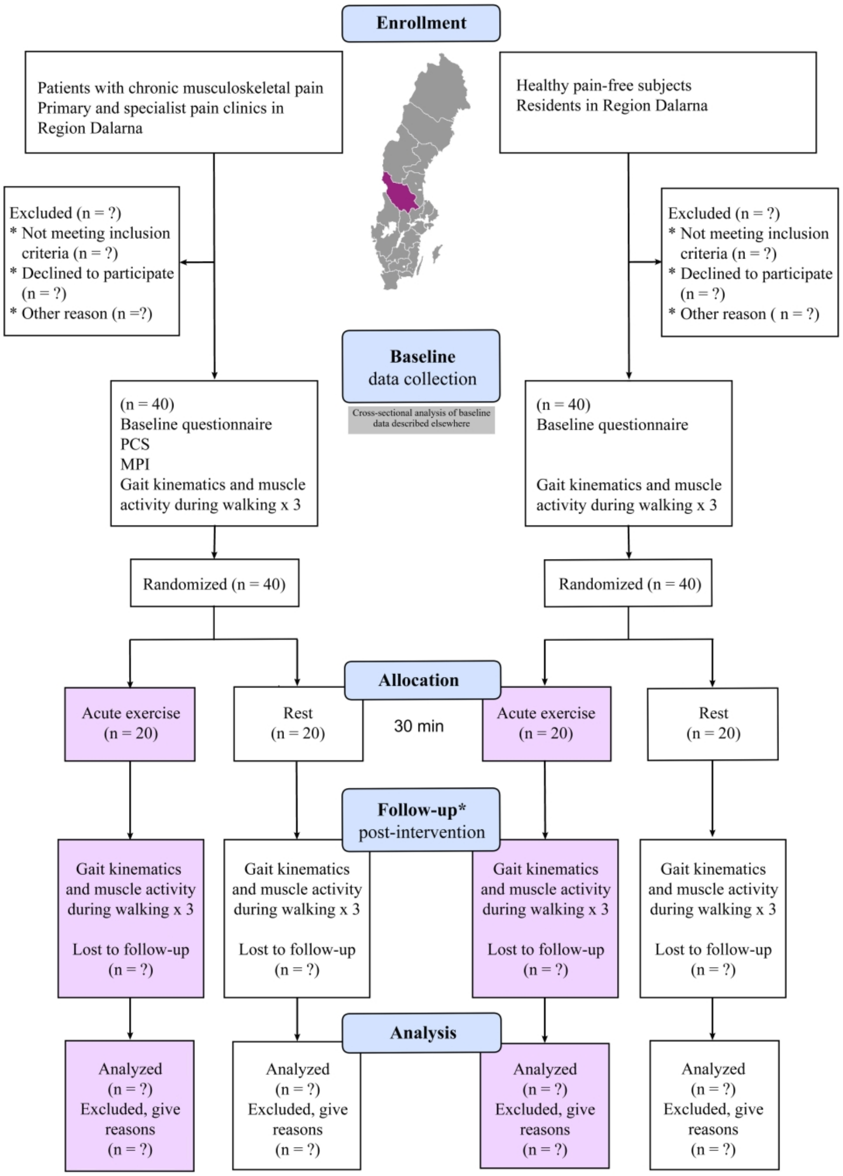

Flowchart

1147x1574mm (96 x 96 DPI)

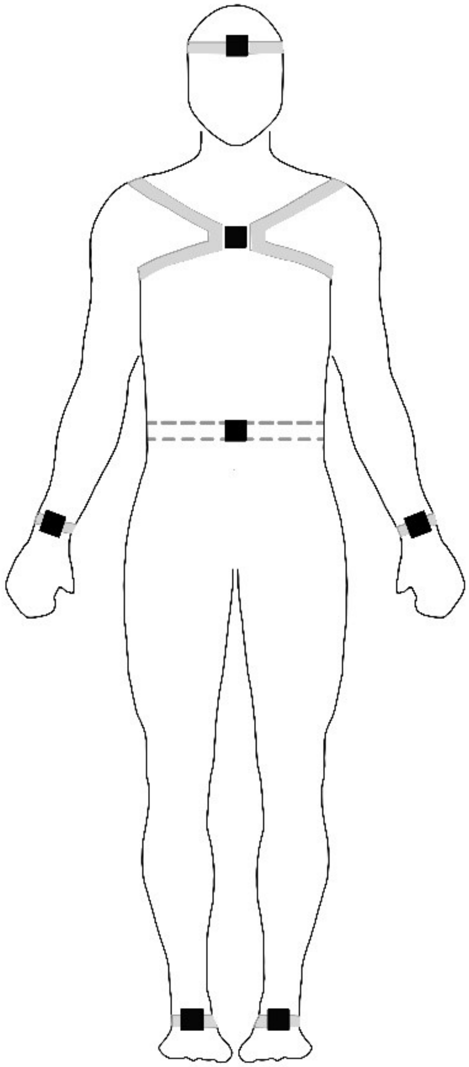

IMU Sensor placement

79x112mm (220 x 220 DPI)

Muscle	EMG electrode locations	Orientation	Illustration
Erector Spinae Neck, bilateral	Placed 20 mm lateral to the median line at the level of C4-C5 spinous process	Vertical	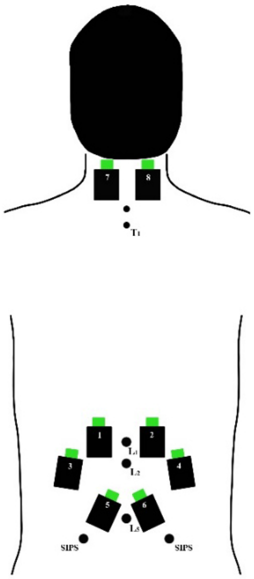
Erector Spinae Longissimus (EL), bilateral	Placed 2 finger widths lateral from the spinous process of L1.	Vertical	
Erector Spinae Iliocostalis (IL), bilateral	Placed 1 finger width medial from a line from the posterior spina iliaca superior to the lowest point of the lower rib, at the level of L2.	Vertical	
Multifidus (MF), bilateral	Placed on and aligned with a line from caudal tip posterior spina iliaca superior to the interspace between L1 and L2 interspace at the level of L5 spinous process (i.e. about 20-30 mm from midline).	Aligned with line, 45° from horizontal	

EMG, electromyography

EMG Sensor placement

390x275mm (79 x 87 DPI)

SPIRIT
STANDARD PROTOCOL ITEMS: RECOMMENDATIONS FOR INTERVENTIONAL TRIALS

Combined SPIRIT 2013 and SPIRIT-PRO 2018 Checklist: Recommended items to address in the clinical trial protocol and related documents by Westergren et al.

Section/item	Item No	Description	Addressed on page number
**Administrative information**			
Title	1	Descriptive title identifying the study design, population, interventions, and, if applicable, trial acronym	1
Trial registration	2a	Trial identifier and registry name. If not yet registered, name of intended registry	2
	2b	All items from the World Health Organization Trial Registration Data Set	2
Protocol version	3	Date and version identifier	2
Funding	4	Sources and types of financial, material, and other support	19
Roles and responsibilities	5a	Names, affiliations, and roles of protocol contributors<br>SPIRIT-PRO<br>Specify the individual(s) responsible for the PRO content of the trial protocol.	1, 19-20
	5b	Name and contact information for the trial sponsor	19 (ej contact info)
	5c	Role of study sponsor and funders, if any, in study design; collection, management, analysis, and interpretation of data; writing of the report; and the decision to submit the report for publication, including whether they will have ultimate authority over any of these activities	19
	5d	Composition, roles, and responsibilities of the coordinating centre, steering committee, endpoint adjudication committee, data management team, and other individuals or groups overseeing the trial, if applicable (see Item 21a for data monitoring committee)	Not applicable
**Introduction**			

For peer review only

Background and rationale	6a	Description of research question and justification for undertaking the trial, including summary of relevant studies (published and unpublished) examining benefits and harms for each intervention<br>SPIRIT-PRO<br>Describe the PRO-specific research question and rationale for PRO assessment and summarize PRO findings in relevant studies	4–6<br>4–6
	6b	Explanation for choice of comparators	5
Objectives	7	Specific objectives or hypotheses<br>SPIRIT-PRO<br>State specific PRO objectives or hypotheses (including relevant PRO concepts/domains).	6 Aims<br>Study hypothesis
Trial design	8	Description of trial design including type of trial (eg, parallel group, crossover, factorial, single group), allocation ratio, and framework (eg, superiority, equivalence, noninferiority, exploratory)	6-7
**Methods: Participants, interventions, and outcomes**			
Study setting	9	Description of study settings (eg, community clinic, academic hospital) and list of countries where data will be collected. Reference to where list of study sites can be obtained	7
Eligibility criteria	10	Inclusion and exclusion criteria for participants. If applicable, eligibility criteria for study centres and individuals who will perform the interventions (eg, surgeons, psychotherapists)<br>SPIRIT-PRO<br>Specify any PRO-specific eligibility criteria (eg, language/reading requirements or prerandomization completion of PRO).<br>If PROs will not be collected from the entire study sample, provide a rationale and describe the method for obtaining the PRO subsample.	8–9<br>9
Interventions	11a	Interventions for each group with sufficient detail to allow replication, including how and when they will be administered	9-14 interventions
	11b	Criteria for discontinuing or modifying allocated interventions for a given trial participant (eg, drug dose change in response to harms, participant request, or improving/worsening disease)	13 (Design) included in ethics approval.
	11c	Strategies to improve adherence to intervention protocols, and any procedures for monitoring adherence (eg, drug tablet return, laboratory tests)	Not Applicable
	11d	Relevant concomitant care and interventions that are permitted or prohibited during the trial	Not Applicable

For peer review only - http://bmjopen.bmj.com/site/about/guidelines.xhtml

Outcomes	12	Primary, secondary, and other outcomes, including the specific measurement variable (eg, systolic blood pressure), analysis metric (eg, change from baseline, final value, time to event), method of aggregation (eg, median, proportion), and time point for each outcome. Explanation of the clinical relevance of chosen efficacy and harm outcomes is strongly recommended	10–13
		SPIRIT-PRO<br>Specify the PRO concepts/domains used to evaluate the intervention (eg, overall health-related quality of life, specific domain, specific symptom) and, for each one, the analysis metric (eg, change from baseline, final value, time to event) and the principal time point or period of interest.	10–13
Participant timeline	13	Time schedule of enrolment, interventions (including any run-ins and washouts), assessments, and visits for participants. A schematic diagram is highly recommended (see Figure)	14-16<br>Figure 1<br>Table 1
		SPIRIT-PRO<br>Include a schedule of PRO assessments, providing a rationale for the time points, and justifying if the initial assessment is not prerandomization. Specify time windows, whether PRO collection is prior to clinical assessments, and, if using multiple questionnaires, whether order of administration will be standardized.	14-16<br>Figure 1<br>Table 1
Sample size	14	Estimated number of participants needed to achieve study objectives and how it was determined, including clinical and statistical assumptions supporting any sample size calculations	7
		SPIRIT-PRO<br>When a PRO is the primary end point, state the required sample size (and how it was determined) and recruitment target (accounting for expected loss to follow-up).<br>If sample size is not established based on the PRO end point, then discuss the power of the principal PRO analyses.	Not Applicable
Recruitment	15	Strategies for achieving adequate participant enrolment to reach target sample size	7

Methods: Assignment of interventions (for controlled trials)

Allocation:

Sequence generation	16a	Method of generating the allocation sequence (eg, computer-generated random numbers), and list of any factors for stratification. To reduce predictability of a random sequence, details of any planned restriction (eg, blocking) should be provided in a separate document that is unavailable to those who enrol participants or assign interventions	8-9
Allocation concealment mechanism	16b	Mechanism of implementing the allocation sequence (eg, central telephone; sequentially numbered, opaque, sealed envelopes), describing any steps to conceal the sequence until interventions are assigned	8-9

For peer review only - http://bmjopen.bmj.com/site/about/guidelines.xhtml

Implementation	16c	Who will generate the allocation sequence, who will enrol participants, and who will assign participants to interventions	8-9
Blinding (masking)	17a	Who will be blinded after assignment to interventions (eg, trial participants, care providers, outcome assessors, data analysts), and how	8-9,
	17b	If blinded, circumstances under which unblinding is permissible, and procedure for revealing a participant's allocated intervention during the trial	NA
**Methods: Data collection, management, and analysis**			
Data collection methods	18a	Plans for assessment and collection of outcome, baseline, and other trial data, including any related processes to promote data quality (eg, duplicate measurements, training of assessors) and a description of study instruments (eg, questionnaires, laboratory tests) along with their reliability and validity, if known. Reference to where data collection forms can be found, if not in the protocol	10–13<br>15–17<br>Table 1
		SPIRIT-PRO<br>Justify the PRO instrument to be used and describe domains, number of items, recall period, and instrument scaling and scoring (eg, range and direction of scores indicating a good or poor outcome). Evidence of PRO instrument measurement properties, interpretation guidelines, and patient acceptability and burden should be provided or cited if available, ideally in the population of interest. State whether the measure will be used in accordance with any user manual and specify and justify deviations if planned.	10–13<br>15–17<br>Table 1
		SPIRIT-PRO<br>Include a data collection plan outlining the permitted mode(s) of administration (eg, paper, telephone, electronic, other) and setting (eg, clinic, home, other).	
		SPIRIT-PRO<br>Specify whether more than 1 language version will be used and state whether translated versions have been developed using currently recommended methods	NA
		SPIRIT-PRO<br>When the trial context requires someone other than a trial participant to answer on his or her behalf (a proxy-reported outcome), state and justify the use of a proxy respondent. Provide or cite evidence of the validity of proxy assessment if available.	NA
	18b	Plans to promote participant retention and complete follow-up, including list of any outcome data to be collected for participants who discontinue or deviate from intervention protocols	NA
		SPIRIT-PRO<br>Specify PRO data collection and management strategies for minimizing avoidable missing data.	
		SPIRIT-PRO<br>Describe the process of PRO assessment for participants who discontinue or deviate from the assigned intervention protocol.	

For peer review only - http://bmjopen.bmj.com/site/about/guidelines.xhtml

Data management	19	Plans for data entry, coding, security, and storage, including any related processes to promote data quality (eg, double data entry; range checks for data values). Reference to where details of data management procedures can be found, if not in the protocol	14
Statistical methods	20a	Statistical methods for analysing primary and secondary outcomes. Reference to where other details of the statistical analysis plan can be found, if not in the protocol	14-15
		SPIRIT-PRO<br>State PRO analysis methods, including any plans for addressing multiplicity/type I (α) error.	14-15
	20b	Methods for any additional analyses (eg, subgroup and adjusted analyses)	14-15
	20c	Definition of analysis population relating to protocol non-adherence (eg, as randomised analysis), and any statistical methods to handle missing data (eg, multiple imputation)	14-15
		SPIRIT-PRO<br>State how missing data will be described and outline the methods for handling missing items or entire assessments (eg, approach to imputation and sensitivity analyses).	14-15
**Methods: Monitoring**			
Data monitoring	21a	Composition of data monitoring committee (DMC); summary of its role and reporting structure; statement of whether it is independent from the sponsor and competing interests; and reference to where further details about its charter can be found, if not in the protocol. Alternatively, an explanation of why a DMC is not needed	19
	21b	Description of any interim analyses and stopping guidelines, including who will have access to these interim results and make the final decision to terminate the trial	Not Applicable
Harms	22	Plans for collecting, assessing, reporting, and managing solicited and spontaneously reported adverse events and other unintended effects of trial interventions or trial conduct	13
		SPIRIT-PRO<br>State whether or not PRO data will be monitored during the study to inform the clinical care of individual trial participants and, if so, how this will be managed in a standardized way. Describe how this process will be explained to participants; eg, in the participant information sheet and consent form.	Not applicable

Auditing	23	Frequency and procedures for auditing trial conduct, if any, and whether the process will be independent from investigators and the sponsor	NA 19
**Ethics and dissemination**			
Research ethics approval	24	Plans for seeking research ethics committee/institutional review board (REC/IRB) approval	2, 19
Protocol amendments	25	Plans for communicating important protocol modifications (eg, changes to eligibility criteria, outcomes, analyses) to relevant parties (eg, investigators, REC/IRBs, trial participants, trial registries, journals, regulators)	14
Consent or assent	26a	Who will obtain informed consent or assent from potential trial participants or authorised surrogates, and how (see Item 32)	11
	26b	Additional consent provisions for collection and use of participant data and biological specimens in ancillary studies, if applicable	NA
Confidentiality	27	How personal information about potential and enrolled participants will be collected, shared, and maintained in order to protect confidentiality before, during, and after the trial	14-15
Declaration of interests	28	Financial and other competing interests for principal investigators for the overall trial and each study site	19
Access to data	29	Statement of who will have access to the final trial dataset, and disclosure of contractual agreements that limit such access for investigators	14-15
Ancillary and post-trial care	30	Provisions, if any, for ancillary and post-trial care, and for compensation to those who suffer harm from trial participation	NA
Dissemination policy	31a	Plans for investigators and sponsor to communicate trial results to participants, healthcare professionals, the public, and other relevant groups (eg, via publication, reporting in results databases, or other data sharing arrangements), including any publication restrictions	19
	31b	Authorship eligibility guidelines and any intended use of professional writers	NA
	31c	Plans, if any, for granting public access to the full protocol, participant-level dataset, and statistical code	20

For peer review

Appendices

Informed consent materials	32	Model consent form and other related documentation given to participants and authorised surrogates	11
Biological specimens	33	Plans for collection, laboratory evaluation, and storage of biological specimens for genetic or molecular analysis in the current trial and for future use in ancillary studies, if applicable	Not Applicable

*It is strongly recommended that this checklist be read in conjunction with the SPIRIT 2013 Explanation & Elaboration for important clarification on the items. Amendments to the protocol should be tracked and dated. The SPIRIT checklist is copyrighted by the SPIRIT Group under the Creative Commons "Attribution-NonCommercial-NoDerivs 3.0 Unported" license.

For peer review only

BMJ Open

Acute exercise as active inference in chronic musculoskeletal pain, effects on gait kinematics and muscular activity in patients and healthy participants – A study protocol for a randomised controlled laboratory trial

Journal:	*BMJ Open*
Manuscript ID	bmjopen-2022-069747.R2
Article Type:	Protocol
Date Submitted by the Author:	16-May-2023
Complete List of Authors:	Westergren, Jens; Dalarna University, School of Health and Welfare, Department of Sports and Medicine<br>Sjöberg, Veronica; Dalarna University, School of Health and Welfare, Department of Sports and Medicine<br>Vixner, Linda; Hogskolan Dalarna, School of Health and Welfare, Department of Sports and Medicine<br>Nyberg, Roger; Dalarna University, School of Technology and Business Studies<br>Conradsson, David; Karolinska Institutet, Department of Neurobiology, Care Sciences and Society, Division of Physiotherapy; Karolinska Universitetssjukhuset, Medical unit Occupational therapy & Physiotherapy, Theme Women's Health and Allied Health Professional<br>Monnier, Andreas; Dalarna University, School of Health and Welfare, Department of Sports and Medicine; Karolinska Institutet, Department of Neurobiology, Care Sciences and Society, Division of Physiotherapy<br>LoMartire, Riccardo; Uppsala University, Department of Research and Education, Center for Clinical Research Dalarna, Region Dalarna<br>Enthoven, Paul; Linköping University, Department of Health, Medicine and Caring Sciences, Pain and Rehabilitation Centre<br>Äng, Björn; Dalarna University, School of Health and Welfare, Department of Sports and Medicine; Uppsala University, Department of Research and Education, Center for Clinical Research Dalarna, Region Dalarna
**Primary Subject Heading**:	Rehabilitation medicine
Secondary Subject Heading:	Sports and exercise medicine
Keywords:	Back pain < ORTHOPAEDIC & TRAUMA SURGERY, PAIN MANAGEMENT, Musculoskeletal disorders < ORTHOPAEDIC & TRAUMA SURGERY

SCHOLARONE™
Manuscripts

BMJ Open

Words in text 3995

Words in abstract 286

Tables 1

Figures 3

Appendices 2 (SPIRIT checklist and model consent)

Acute exercise as active inference in chronic musculoskeletal pain, effects on gait kinematics and muscular activity in patients and healthy participants – A study protocol for a randomised controlled laboratory trial

Jens Westergren,[1] Veronica Sjöberg,[1] Linda Vixner,[1,] Roger G Nyberg,[1,7] David Moulaee Conradsson,[2,3] Andreas Monnier,[1,2,8] Riccardo LoMartire,[6] Paul Enthoven,[4,5] Björn O Äng.[1,2,6]

[1]Department of Sport and Medicine, School of Health and Welfare, Dalarna University, Falun, Sweden

[2]Department of Neurobiology, Care Sciences and Society, Division of Physiotherapy, Karolinska Institutet, Huddinge, Sweden

[3]Medical unit Occupational therapy & Physiotherapy, Theme Women's Health and Allied Health Professional, Karolinska University Hospital, Stockholm, Sweden

[4]Department of Health, Medicine and Caring Sciences, Physiotherapy, Linköping University, Linköping, Sweden

[5]Pain and Rehabilitation Centre, and Department of Health, Medicine and Caring Sciences, Linköping University, Linköping, Sweden

[6]Department of Research and Education, Center for Clinical Research Dalarna – Uppsala University, Region Dalarna, Falun, Sweden

[7]School of Technology and Business Studies, Dalarna University, Falun, Sweden

[8] Military Academy Karlberg, Swedish Armed Forces, Solna, Sweden

Corresponding author:

For peer review only - http://bmjopen.bmj.com/site/about/guidelines.xhtml

29 Jens Westergren
30 Department of Sport and Medicine, School of Health and Welfare, Dalarna University, Falun,
31 Sweden
32 E-Mail: jws@du.se

33

For peer review only

For peer review only - http://bmjopen.bmj.com/site/about/guidelines.xhtml

ABSTRACT

Introduction

Chronic musculoskeletal pain is a highly prevalent, complex, and distressing condition that may negatively affect all domains of life. In view of an active inference framework, and resting on the concept of allostasis, human movement per se becomes a prerequisite for health and wellbeing while chronic pain becomes a sign of a system unable to attenuate an allostatic load. Previous studies on different subgroups of chronic pain conditions have demonstrated alterations in gait kinematics and muscle activity, indicating shared disturbances in the motor system from long-term allostatic load. We hypothesize that such alterations exist in heterogenous populations with chronic musculoskeletal pain, and that exposure to acute and controlled exercise may attenuate these alterations. Therefore, the main aim of this study is to investigate the acute effects of exercise on gait kinematics and activity of the back and neck muscles during diverse walking conditions in patients with chronic musculoskeletal pain compared to a reference sample consisting of healthy participants.

Methods and analysis

This two-sample two-armed parallel randomised controlled laboratory trial will include 40 participants with chronic musculoskeletal pain (> 3 months) and 40 healthy participants. Participants will be randomly allocated to either 30 min of aerobic exercise or rest. Primary outcomes are gait kinematics (walking speed, step frequency, stride length, lumbar rotation, gait stability) and muscular activity (spatial and temporal) of the back and neck during diverse walking conditions. Secondary outcomes are variability of gait kinematics and muscle activity and subjective pain ratings assessed regularly during the trial.

For peer review only - http://bmjopen.bmj.com/site/about/guidelines.xhtml

Ethics and dissemination

The study has been approved by the Regional Ethics Review Board in Uppsala, Sweden (#2018/307/308). Findings will be disseminated via conference presentations, publications in peer-reviewed journals, and engagement with patient support groups and clinicians.

Trial Registration: ClinicalTrials.gov; NCT03882333, March 20, 2019.

Keywords:

Chronic Musculoskeletal Pain, Active Inference, Acute Aerobic Exercise, Gait Kinematics, Electromyography

For peer review only - http://bmjopen.bmj.com/site/about/guidelines.xhtml

Strengths and limitations of this study

- To our knowledge, this will be the first randomised controlled laboratory trial investigating the acute effects of aerobic exercise, as active inference, on gait kinematics and muscular activity in a heterogenous group of patients with chronic musculoskeletal pain.
- As long-term aerobic exercise is ultimately the result of regular sessions of aerobic exercise, studying effects induced by a single session of aerobic exercise will provide important insights into exercise and its role in the rehabilitation of chronic pain conditions.
- The study will incorporate objective measures of gait kinematics and muscle activity from continuous overground walking by utilizing modern wireless sensors, thus replicating real world conditions of functional movement.
- While complete blinding is not possible post intervention, due to the nature of exercise, care will be taken to minimize investigator bias by using automated recording protocols.

Based on protocol version 1.0; May 12, 2020.

For peer review only

For peer review only - http://bmjopen.bmj.com/site/about/guidelines.xhtml

INTRODUCTION

Chronic musculoskeletal pain is a highly prevalent and complex condition that continues to challenge clinicians and academics and as such constitutes a major health and wellbeing burden.[1 2] The International Association for the Study of Pain (IASP) defines chronic pain as "pain which has persisted beyond normal tissue healing time", which, in the absence of other factors, is a pain condition lasting for more than three months.[3] A condition with chronic musculoskeletal pain may be referred to as chronic primary pain[4] and should be seen as a disease in its own right.[5] Chronic musculoskeletal pain can negatively affect physical movement and functioning, sleep, social interaction, quality of life, and work ability,[6] in essence all domains of life.

In the light of an active inference framework,[7-9] based on the free energy principle proposed by Friston[10 11] and resting on the concept of allostasis,[12] any living system becomes a model of the world it inhabits. Then, through the process of active inference it attempts to maximize the proof of its own existence (chances of survival) by minimizing prediction errors (variational free energy) through perception and action. The physiological concept of allostasis can be described as achieving stability through change,[12] in which the rate of change influences the process of adaption. In addition, living things may be defined by their ability to move autonomously, be it of bodies, limbs, or cells, making movement a key driver of allostasis through sensory palpation of the world. In view of this, human movement, from cell respiration to locomotion, may constitute the major source of active inference upon which the organism builds and maintains its model, making human movement of all sorts pertinent to long-term health and prosperity by facilitating the changes that are needed for stability.

For peer review only - http://bmjopen.bmj.com/site/about/guidelines.xhtml

Building on this notion, in a healthy context acute pain can be viewed as a precision signal for learning and control, with the main function being motivational to direct both short- and long-term motor behaviour away from harm.[13] Chronic musculoskeletal pain on the other hand, is suggestive of a living system in distress and may be explained by the creation and maintenance of abnormal priors that are afforded abnormal attention in a self-evidencing system.[7 14]

This reasoning is – we believe – in line with earlier theories such as the body-self neuromatrix model,[15] the theory of Moving differently in pain,[16] and effectively aligning with concepts such as sensitization,[17 18] and ultimately depression[19] within chronic pain populations, as well as recent theory on active interoceptive inference.[20] Then chronic pain can also be viewed as the consequence of an allostatic overload from unresolved prediction errors of the systems lower level predictions about its internal and external milieu.[17 19 21-24] These prediction errors in lower level predictions may manifest themselves at higher levels as fear avoidance behaviour of movement[25] and intolerance of uncertainty,[26] which lead to the further reduction of physical activity.[27-29] This relative sensory deprivation[30 31] that is induced by sedentary behaviour may disable self-organisation normally driven through allostasis, leading to aberrant neuro-signatures (models) of painful states. In essence, chronic musculoskeletal pain becomes a pathophysiology with a self-fulfilling hypothesis of pain as the systems only solution to minimize prediction errors. Building on this notion, physical activity and exercise, or simply human movement, can be viewed as active inference and as such may facilitate a return to allostasis and less painful future states by promoting the flexible reassignment of precision weighting that alters the individual's prediction about their body and their world.[32] This implies that adding physical activity or exercise to a

For peer review only - http://bmjopen.bmj.com/site/about/guidelines.xhtml

rehabilitation program, may simply be reintroducing something essential that was engineered away generations ago.

From this active inference view of chronic pain, it is understandable that a biopsychosocial approach has gained traction in the treatment of chronic pain conditions,[6,33-35] since such interdisciplinary treatment delivered by a team of allied health professionals involves a combination of physical activity and exercise, psychological interventions, pharmaceutical treatments, and patient education all acting as active inference[33] enabling a return to less painful states.

Given the fact that the musculoskeletal system is crucial to human locomotion or functional movement, a large body of research has focused on the effects of chronic pain on functional movement. There is now moderate to strong evidence that individuals with persistent low back pain demonstrate differences in gait kinematics,[34] that individuals with non-specific low back pain demonstrate impaired postural control when standing,[35] and that individuals diagnosed with fibromyalgia demonstrate an altered gait compared to healthy individuals.[36] Neuroimaging studies have reported functional brain changes related to sensorimotor impairment in individuals with low back pain,[37] irritable bowel syndrome,[14] fibromyalgia,[38] and migraine,[39] suggestive of shared disturbances of the motor system across different diagnoses. The overall finding is that persons with chronic pain conditions walk slower, with a lower step frequency, shorter step-length, and with a greater amplitude of back muscle activity.[34 36]

However, based on the notion of a shared disturbance it is currently not known if the altered gait patterns identified in specific sub-groups of chronic pain patients may generalize to wider

For peer review only - http://bmjopen.bmj.com/site/about/guidelines.xhtml

populations of chronic musculoskeletal pain patients and if they could be used as markers of functional movement impairment in these populations. There is a paucity in how exercise, viewed as active inference can have an acute effect on the motor control of walking in humans, especially in adaptions to perturbations such as faster walking speeds or dual tasks and if this is related to long-term sedentary behaviour.

Therefore, the main aim of this study is to investigate the acute effects of exercise as active inference on gait kinematics and activity of the back and neck muscles during diverse walking conditions in patients with chronic musculoskeletal pain compared to a reference sample consisting of participants without chronic musculoskeletal pain.

Our hypothesis is that a wide sample with chronic musculoskeletal pain will walk at lower speeds with lower step frequency and stride lengths but with increased muscle activity in the back and neck and that these differences will increase with task difficulty and begin to attenuate acutely by exercise.

MATERIALS AND METHODS

Trial design

The study consists of a two-sample two-armed parallel randomised controlled laboratory trial (RCT) that will be conducted at Dalarna University, Sweden (Figure 1). The main sample consists of patients with chronic pain who will be randomised into one of two arms: an intervention group performing a single session of acute aerobic exercise (30 min stationary cycling) or a resting control group. The reference sample consists of participants without chronic musculoskeletal pain who will follow a similar RCT procedure, with the purpose being to investigate whether the acute response to exercise is unique for patients with chronic

For peer review only

For peer review only - http://bmjopen.bmj.com/site/about/guidelines.xhtml

pain as our hypothesis proposes, or if it is just a general effect that would be seen in any population. The trial will comply with Consolidated Standards of Reporting Trials (CONSORT) and Standard Protocol Items: Recommendations for Interventional Trials guidelines with Patient Reported Outcomes (SPIRIT).[40-42] A completed SPIRIT checklist for the trial can be found in appendix 1. Data will be collected between January 2019 and May 2021.

For our primary outcome, a total estimated sample size of n = 80 (n =20 in each of the four randomised groups) was determined based on previous studies using similar design and wearable technology. [43 44] A supplementary power calculation was conducted using normalized right-side Erector Spinae Longissimus back muscle activity collected from the initial 20 participants to confirm the adequacy of the sample size. The analysis confirmed that a total sample size of n = 80 is required to achieve a statistical power of 80% at a significance level of α = 0.05 (two tailed) to detect a 25% difference between patients and participants without musculoskeletal pain.

Please insert Figure 1 here

Participants and setting

This trial includes 80 participants. Patients (n=40) that visit primary and specialist pain clinics in Region Dalarna for evaluation or treatment of chronic musculoskeletal pain (> 3 months), as well as an age- and gender-matched convenience sample of participants without

For peer review only - http://bmjopen.bmj.com/site/about/guidelines.xhtml

musculoskeletal pain (n=40) will be simultaneously recruited. The physiotherapists treating or evaluating potential participants at the cooperating clinics will act as liaisons to inform patients of the study and those interested in participation will be asked to provide their contact information. Researchers will then contact all potential participants by telephone and e-mail prior to the laboratory visit, to perform a preliminary screening of eligibility criteria before scheduling a laboratory visit.

Eligibility criteria

Participants aged 18-67 years will be included. Patients will be in assessment and/or treatment of chronic (>3 months) musculoskeletal (neck and/or low back) pain or widespread pain in primary or specialist pain care in Region Dalarna. Exclusion criteria: 1) Any co-existing medical condition that would restrict safely participating in the exercise intervention. 2) Previous spinal surgery, 3) Inability to perform unsupported indoor walking, 4) Inadequate Swedish or cognitive ability to provide consent and understand information and instructions, 5) Pregnant in second or third trimester or childbirth in the last three months, 6) Allergies to adhesive tape used for sensor application, 7) Major hearing impairment effecting performance of auditory 1-back test, 7) Pain caused by malignancies or systemic diseases, or localized pain in lower extremities only.

Randomisation, allocation concealment and blinding

Participants will be randomly assigned to either the intervention or control group at a 1:1 allocation ratio, using stratified block randomization with a fixed block size of six within the chronic musculoskeletal pain and no chronic pain strata. Allocation information is kept in opaque sealed envelopes and will not be disclosed to participants or researchers until all baseline walk trials are completed. All data collection will be automated in pre-programmed

For peer review only

For peer review only - http://bmjopen.bmj.com/site/about/guidelines.xhtml

sequences and participant instructions read verbatim to minimize any investigator bias influencing participants' behaviour. Participants will not be informed of the hypotheses of the study, and the researcher performing the data analyses will be blinded to group allocation.

Interventions

The acute aerobic exercise intervention will consist of one 20-minute cycling interval preceded by a five-minute warm-up and followed by a five-minute cool-down (total duration of 30 minutes) and will be performed on a stationary electromagnetically braked cycle ergometer (Schoberer Rad Messtechnik SRM, Jülich, Germany). Self-selected exercise intensity will be guided to moderate intensity by using Borg Ratings of Perceived Exertion (RPE) scale with a target RPE of 13-15 (somewhat hard/hard).[45] Borg RPE and self-rated pain on VAS (0-100mm) will be recorded every five-minutes during the exercise session. Power output, cadence, and heart rate will be recorded at 2 Hz. Participants will be able to freely regulate cadence and resistance to maintain the target RPE of 13-15, by means of a 14-increment mechanical gear shifter located on the handlebars. The ergometer is equipped with an upright handlebar, flat pedals and a wide padded saddle with an extra gel cover to minimize sitting discomfort.

Participants allocated to the control groups will rest for the equivalent period of 30 minutes in a self-selected comfortable resting position accompanied by staff.

For peer review only - http://bmjopen.bmj.com/site/about/guidelines.xhtml

Outcome measures

The primary outcomes (detailed in table 1) are kinematic measures of human movement and muscle activity that will be measured while participants are walking using the following three gait conditions: 1) self-selected habitual gait speed, 2) brisk gait speed, and 3) self-selected habitual gait speed with a concurrent cognitive task. Our primary kinematic outcomes are gait speed, step frequency, stride length, lumbar rotation, and gait stability, while primary outcomes for the muscle activity domain will be mean normalized EMG amplitude averaged over aggregated gait cycles.

Our secondary outcome measures are variability of gait kinematics and muscle activity and self-rated current pain intensity using a horizontal 100-mm line Visual Analogue Scale (VAS) with the statement 'no pain at all' at the extreme left, and 'the worst possible pain' at the extreme right (chronic pain sample only).[46 47]

Table 1. Outcomes and assessments

			Baseline	Intervention	Follow-up
Baseline measures	Variable or assessment	Data Source			
Participant characteristics	Age, sex, body mass index (weight, height), educational level, work status, physical activity.	SAQ+ PT	X		
Pain catastrophizing †	Pain Catastrophizing Scale (PCS)	SAQ	X		
Effects on everyday life †	Multidimensional Pain Inventory (MPI)	SAQ	X		
Primary outcome measures					
**Gait kinematics**<br>- Gait speed (m/s)<br>- Step frequency (steps min-1)<br>- Stride length (cm)<br>- Lumbar rotation (°)<br>- Lateral step variability (three consecutive steps, the perpendicular deviation of the middle foot placement from the line connecting the first and the third step)<br><br>**Back and neck muscle activity**<br>- Normalized mean muscle activity of all completed gait cycles per trial<br>- Back and neck normalized muscle activity at each 10 percentile of gait cycle	Gait kinematics with IMU-based sensors and wireless surface EMG, aggregated from minimum 50 gait cycles<br>-Self -selected habitual gait speed<br>- Brisk gait speed‡<br>- Self-selected habitual gait speed with concurrent cognitive task (1-back test) ‡	PT	X		X

For peer review only - http://bmjopen.bmj.com/site/about/guidelines.xhtml

Secondary outcome measures					
**Variability of gait kinematics**<br>- Gait speed (m/s)<br>- Step frequency (steps min-1)<br>- Stride length (cm)<br>- Lumbar rotation (°)<br>- Lateral step variability<br><br>**Variability of muscle activity**<br>- Normalized mean per trial<br>- Balance of left/right at each 10 percentile of gait cycle<br>- Back and neck normalized during stance and swing phase<br>- Number of muscle activity spikes (>100% normalized per complete gait cycle)	Non-linear analysis of gait kinematics with IMU-based sensors and wireless surface EMG, aggregated from minimum 50 gait cycles<br>-Self -selected habitual gait speed<br>- Brisk gait speed‡<br>- Self-selected habitual gait speed with concurrent cognitive task (1-back test) ‡	PT	X		X
- Pain intensity†	VAS (0-100)	SAQ	X	X	X

Data source: Self-administered questionnaire (SAQ), physical testing (PT).

VAS, Visual Analogue Scale; administered after every gait test. RPE, Rating of Perceived Exertion; every 5 minutes during the intervention; IMU, Inertial Monitoring Unit; EMG, Electromyography.

† Only measured in patients with chronic musculoskeletal pain

‡ Brisk gait speed and self-selected gait speed with the 1-back test will be performed in random order

For peer review only - http://bmjopen.bmj.com/site/about/guidelines.xhtml

Trial procedure, instruments, and data collection

The entire trial procedure at the laboratory is expected to take approximately three hours and will be supervised by two researchers. Upon arrival at the laboratory, eligibility screening, presentation of the study procedures, and collection of informed consent (Appendix 2) will be performed. Participants will complete the following questionnaires before commencing any testing: 1) Participant characteristics (age, sex, educational level, occupational status, pain locations, pain duration) including two validated items with questions about physical activity.[48] Participants with chronic musculoskeletal pain will also complete the following two questionnaires relating to pain experiences: 2) The Pain Catastrophizing Scale (PCS)[49] which contains 13 items regarding participants' thoughts and feelings associated with pain, with a total score ranging from 0-52, with higher scores representing higher pain catastrophizing, and 3) The West Haven-Yale Multidimensional Pain Inventory (MPI), which is a questionnaire that measures the psychosocial, cognitive, and behavioural effects of chronic pain.[50-53]

A researcher will then record participant body height using a stadiometer (Harpenden Stadiometer, Holtain Limited, Crosswell, UK) and body weight using a weighing scale (Midrics, Sartorius AG, Goettingen, Germany). Participants will be introduced to the 1-back test[54] and listen to a 30-second practice sequence, then two 30-second 1-back baseline tests will be recorded with participants in a seated position. The 1-back test will be performed with a pair of minimally intrusive wireless handsfree on-ear headphones connected via Bluetooth to a PC using Audacity Software v. 2.3.0. Participants will listen to an audio file consisting of a randomized number sequence and will be instructed to respond as quickly and accurately as possible to audio prompts, repeating the digit before the last one in the consecutive sequence during the entire 1-back walking trial. All responses will be recorded.

For peer review only - http://bmjopen.bmj.com/site/about/guidelines.xhtml

After this, seven wireless inertial monitoring unit (IMU) sensors (OPAL, APDM INC, OR, USA) incorporating a 3-axis- gyroscope, accelerometer, and magnetometer will be applied on participants' limbs using elastic straps as specified in Figure 2. Gait kinematics will be streamed and logged wirelessly from each sensor at 128Hz during all trials and will be automatically processed via Moveo Explorer™ software to extract gait variables specified as primary or secondary outcomes according to Table 1.[55] Participants will be given standardized instructions prior to starting each gait trial in order to comply with the initial calibration procedure described by the manufacturer.

Please insert Figure 2 here

Wireless electromyography (EMG) sensors (Delsys Trigno, Delsys Inc., Boston, MA, USA) will be positioned according to anatomical landmarks detailed in Figure 3, as per SENIAM guidelines[56 57] and data will be recorded at 1111 Hz. Skin will be shaved and cleansed with a chlorhexidine digluconate ethanol cutaneous solution (Klorhexidinsprit 5mg/ml, Fresenius Kabi AB, Uppsala, Sweden). To obtain normalized (reference) muscle activity, each participant will perform four standardized submaximal standing trunk flexion (hip flexion to 45° from neutral standing position) muscle contraction tests, interspersed by a 60-second rest. EMG sensors will be removed after baseline measurements and reapplied prior to the follow-up walking trials. Before removal, sensor positions will be marked with a marker pen to facilitate accurate reapplication.

Please insert Figure 3 here

For peer review only - http://bmjopen.bmj.com/site/about/guidelines.xhtml

324 All gait trials will be performed indoors in a continuous fashion on a 15-meter standardised
325 walkway with tape markings on the floor indicating starting and 180° turning points. Trials
326 will have a minimum duration of two minutes and finish when participants cross the start line
327 with a time exceeding two minutes. Participants will initially perform a familiarization trial at
328 a self-selected habitual speed followed by a short rest if needed, to avoid any accumulated
329 pain or fatigue. After this, participants will proceed to walk the 15-meter walkway under the
330 three different conditions described above: 1) an initial self-selected habitual gait speed, 2) a
331 brisk gait speed, and 3) a self-selected habitual gait speed with a concurrent cognitive task (1-
332 back test). The order of performance of the brisk and 1-back walking conditions (2 or 3) will
333 be randomised and the allocation will be kept concealed from the participants until the initial
334 condition with self-selected habitual gait speed has been performed. For the brisk walking
335 condition, participants will be instructed to walk faster than they habitually do, as they
336 imagine themselves doing when slightly late for a bus they want to catch. The self-rated pain
337 intensity (VAS) of participants with chronic musculoskeletal pain will be recorded after each
338 walk trial. The risk of complications due to measurements or interventions is considered to be
339 low, but any adverse or serious adverse events will be logged and reported as well as covered
340 by laboratory insurance.

341

342 **Data management**

343 Participant questionnaires will be checked for completeness and congruity before data is
344 entered into the database. Data will be stored on secure university servers and in locked
345 cabinets only available to the research team. Gait kinematics will be aggregated for all valid
346 gait cycles of each gait condition. For each individual, a minimum of 50 gait cycles will be
347 used and aggregated prior to analysis.[58] Back and neck muscle activity (root-mean square,

For peer review only - http://bmjopen.bmj.com/site/about/guidelines.xhtml

RMS) synchronized to gait cycles and aggregated from each gait condition will be normalized as percentage RMS against reference contractions.

Statistical analysis

Participant characteristics will be reported using descriptive statistics. The statistical analyses will be performed on an intention-to-treat basis and potential differences between the intervention groups and the control groups will be analysed. Analysis will primarily be conducted with variance analysis with respect to both data quality and distribution (data assumptions) and with the addition of repeated measurements where applicable. Log transformations of data will be made as appropriate. Data will be presented with means and variances. To explore the magnitude of the effect of interventions, effect sizes will be calculated.[59] Lastly, non-linear analysis using Gait Evaluation Differential Entropy Method (GEDEM)[60] will be used to assess variability of gait kinematic and muscular activity.[60-62]

Ethics and dissemination

The study has been approved by the Regional Ethics Review Board in Uppsala, Sweden (#2018/307/308). Findings will be disseminated via conference presentations, publications in peer-reviewed journals, and engagement with patient support groups and clinicians.

Patient and public involvement

The burden of the study procedures has been assessed by patients during preliminary pilot work, primarily to assess the following areas: the feasibility of the cycling exercise intervention, the baseline questionnaires, and the instrumentation to capture gait kinematics and EMG-data. Study participants will be invited to a briefing where the results of the study

For peer review only - http://bmjopen.bmj.com/site/about/guidelines.xhtml

will be presented. In addition, study participants will be given a summary of the results written in Swedish. Results will be presented at the annual meeting of the Swedish National Registry for Chronic Pain Rehabilitation where patient groups are represented.

DISCUSSION

To our knowledge, the current study is the first laboratory RCT to investigate the acute effect of aerobic exercise on gait kinematics and muscle activity in participants with chronic musculoskeletal pain compared to reference participants from a perspective of exercise as active inference. Our two-sample two-armed parallel design allows for a cross comparison of effects in patients with chronic musculoskeletal pain and healthy participants that will follow the same study protocol and will contribute to our understanding of how chronic musculoskeletal pain manifests on measures of human motor behaviour and how exercise can influence this in a rehabilitation setting. Knowledge of how gait kinematics and back and neck muscular activity may change during different gait conditions and from aerobic exercise may contribute to the understanding of how exercise therapy influences our generative models in the process attenuates allostatic load through active inference. The use of modern, miniaturized, and completely wireless sensors during overground walking replicates real world conditions, hence strengthening the ecological validity of our results. Objective measurements of movement and muscular activity during diverse walking conditions have been chosen as primary outcomes since such a methodology can both uniquely and sensitively show and quantify deviating activation patterns as validly reflected in every day functional movements and commonly performed physical activities.

For peer review only

For peer review only - http://bmjopen.bmj.com/site/about/guidelines.xhtml

Participants

Selection bias is difficult to completely overcome in exercise intervention studies as volunteering participants will likely be positively biased towards exercise in general and motivation to participate in an exercise intervention is also likely influenced by the severity of symptoms experienced when asked to volunteer for the study. We will compare and validate our sample baseline characteristics with a new nationwide large database including 60,000 patients with chronic pain.[63]

Concerning our reference participants without chronic musculoskeletal pain, we aim to create a sample that is sex and age matched with our chronic musculoskeletal pain sample. It will serve as a reference sample for all our outcomes – can our hypothesis on the acute effects of exercise in our sample with chronic pain be verified or may unexpected results emerge when healthy participants are subjected to the same intervention?

Intervention

Viewing this exercise intervention as active inference will hopefully add knowledge on how acute movement through exercise may promote an individual's predictions about their body and their world by creating a platform for belief updating. Our use of a cycling modality with a rather different muscle recruitment and joint loading pattern compared to walking may be beneficial, especially since stationary cycling may be more palatable to participants than running at the same intensity as it may lead to an undesired level of fatigue, fear, or increased pain.

In addition, the aerobic exercise intervention in the current study reflects recommendations in exercise guidelines and allows an easy progression of rehabilitation in terms of both intensity

For peer review only - http://bmjopen.bmj.com/site/about/guidelines.xhtml

and duration.[64] Since a bicycle can be used for therapy, recreation, and transportation, there are opportunities for health-care professionals to facilitate changes in lifestyle that have the potential to mitigate morbidity and function in patients with chronic pain,[65] simultaneously reducing the risks and impact of common co-morbidities.

Outcomes

With the foundation of this project being objective measures of overground walking, it adheres well to the Initiative on Methods, Measurement and Pain Assessment in Clinical Trials (IMMPACT), which recommends that all chronic-pain trials should include measures of physical functioning.[47] This project is expected to result in increased knowledge of how acute exercise affects subsequent functional tasks, here measured during different walking conditions.

Potential impact

Our proposed project will deepen our understanding on the topic of alterations in functional movement in chronic musculoskeletal pain conditions.

Perspective

Results from the current study may help us reach a better understanding of how acute aerobic exercise acts as active inference to attenuate chronic pain by facilitating allostasis. This knowledge will help us formulate better questions as to how generative models in chronic pain conditions are formed, maintained, and updated through movement, or lack thereof.

For peer review only - http://bmjopen.bmj.com/site/about/guidelines.xhtml

Author Contributions

All authors meet the ICMJE criteria for authorship. JW, VS, BÄ, and LV take responsibility for the integrity of the study. All authors were involved in the study's concept and design. JW and VS will be involved in the acquisition of data. JW, PE, AM, RLM, DMC, RGN, and BÄ were involved in drafting and revising the article critically for important intellectual content. All authors were involved in manuscript revision and read and approved the final version to be published.

Funding

This work is supported by the Swedish Research Council for Health (2015-02512), Working Life and Welfare (FORTE; 2017-00177), and Dalarna University (N/A), Falun, Sweden. The funders had no role in study design and will have no role in any part of the study or reporting of the results.

Competing interests

None declared.

Patient consent for publication

Not required.

Ethics approval

The study has been approved by the Regional Ethics Review Board in Uppsala, Sweden; approval ID 2018/307/308.

For peer review only - http://bmjopen.bmj.com/site/about/guidelines.xhtml

Data sharing statement

Not applicable

Open access

This is an open access article distributed in accordance with the Creative Commons Attribution Non-Commercial (CC BY-NC 4.0) license, which permits others to distribute, remix, adapt, build upon non-commercially, and license their derivative works on different terms, provided the original work is properly cited, appropriate credit is given, any changes made indicated, and the use is non-commercial.

See: http://creativecommons.org/licenses/by-nc/4.0/.

FIGURE CAPTIONS:

Fig. 1. Participant flowchart.
PCS, Pain Catastrophizing Scale; MPI, Multidimensional Pain Inventory.

Figure 2. IMU-sensor placement on the forehead, sternum, lumbar spine, wrists, and feet. IMU, Inertial monitoring unit. Adapted with permission by APDM, INC.

Figure 3. Overview wireless electrode placement for surface EMG recording of the trunk and neck musculature according to SENIAM guidelines (http://www.seniam.org/)

For peer review only

REFERENCES

1. Leadley RM, Armstrong N, Lee YC, et al. Chronic diseases in the European Union: the prevalence and health cost implications of chronic pain. *Journal of pain & palliative care pharmacotherapy* 2012;26(4):310-25. doi: 10.3109/15360288.2012.736933 [published Online First: 2012/12/12]
2. Global Burden of Disease Study Collaborators. Global, regional, and national incidence, prevalence, and years lived with disability for 328 diseases and injuries for 195 countries, 1990-2016: a systematic analysis for the Global Burden of Disease Study 2016. *Lancet* 2017;390(10100):1211-59. doi: 10.1016/S0140-6736(17)32154-2 [published Online First: 2017/09/19]
3. Raja SN, Carr DB, Cohen M, et al. The revised International Association for the Study of Pain definition of pain: concepts, challenges, and compromises. *Pain* 2020;161(9):1976-82. doi: 10.1097/j.pain.0000000000001939 [published Online First: 2020/07/23]
4. Nicholas M, Vlaeyen JWS, Rief W, et al. The IASP classification of chronic pain for ICD-11: chronic primary pain. *Pain* 2019;160(1):28-37. doi: 10.1097/j.pain.0000000000001390 [published Online First: 2018/12/27]
5. Niv D, Devor M. Chronic pain as a disease in its own right. *Pain Pract* 2004;4(3):179-81. doi: 10.1111/j.1533-2500.2004.04301.x
6. Ahlberg M. Rehabilitering vid långvarig smärta: En systematisk litteraturöversikt (SBU; Swedish agency for health technology assessment and assessment of social services). 2010
7. De Ridder D, Adhia D, Vanneste S. The anatomy of pain and suffering in the brain and its clinical implications. *Neuroscience & Biobehavioral Reviews* 2021;130:125-46. doi: https://doi.org/10.1016/j.neubiorev.2021.08.013
8. Ongaro G, Kaptchuk TJ. Symptom perception, placebo effects, and the Bayesian brain. *Pain* 2019;160(1):1-4. doi: 10.1097/j.pain.0000000000001367
9. Pezzulo G, Parr T, Friston K. The evolution of brain architectures for predictive coding and active inference. *Philos Trans R Soc Lond B Biol Sci* 2022;377(1844):20200531. doi: 10.1098/rstb.2020.0531 [published Online First: 20211227]
10. Friston K. The history of the future of the Bayesian brain. *Neuroimage* 2012;62(2):1230-3. doi: 10.1016/j.neuroimage.2011.10.004 [published Online First: 2011/10/26]
11. Friston K. Life as we know it. *J R Soc Interface* 2013;10(86):20130475. doi: 10.1098/rsif.2013.0475 [published Online First: 20130703]
12. Sterling P, Eyer J. Handbook of Life Stress, Cognition and Health Edited by S. Fisher and J. Reason Copyright 1988 John Wiley 8. Sons, 1988.
13. Seymour B. Pain: A Precision Signal for Reinforcement Learning and Control. *Neuron* 2019;101(6):1029-41. doi: 10.1016/j.neuron.2019.01.055
14. Nisticò V, Rossi RE, D'Arrigo AM, et al. Functional neuroimaging in Irritable Bowel Syndrome: a systematic review highlights common brain alterations with Functional Movement Disorders. *J Neurogastroenterol Motil* 2022 doi: 10.5056/jnm21079 [published Online First: 20220222]
15. Melzack R, Katz J. Pain. *Wiley Interdisciplinary Reviews: Cognitive Science* 2013;4(1):1-15. doi: 10.1002/wcs.1201

For peer review only - http://bmjopen.bmj.com/site/about/guidelines.xhtml

16. Hodges PW, Tucker K. Moving differently in pain: a new theory to explain the adaptation to pain. *Pain* 2011;152(3 Suppl):S90-8. doi: 10.1016/j.pain.2010.10.020 [published Online First: 2010/11/23]
17. Wallden M, Nijs J. Before & beyond the pain – Allostatic load, central sensitivity and their role in health and function. *Journal of Bodywork and Movement Therapies* 2021;27:388-92. doi: https://doi.org/10.1016/j.jbmt.2021.04.003
18. Wallden M, Nijs J. Applying the understanding of central sensitization in practice. *Journal of Bodywork and Movement Therapies* 2021;27:723-30. doi: 10.1016/j.jbmt.2021.04.004
19. Arnaldo I, Corcoran AW, Friston KJ, et al. Stress and its sequelae: An active inference account of the etiological pathway from allostatic overload to depression. *Neuroscience and biobehavioral reviews* 2022;135:104590. doi: 10.1016/j.neubiorev.2022.104590 [published Online First: 20220218]
20. Fermin ASR, Friston K, Yamawaki S. An insula hierarchical network architecture for active interoceptive inference. *R Soc Open Sci* 2022;9(6):220226. doi: 10.1098/rsos.220226 [published Online First: 20220629]
21. Craig AD. How do you feel? Interoception: the sense of the physiological condition of the body. *Nature Reviews Neuroscience* 2002;3(8):655-66. doi: 10.1038/nrn894
22. Craig AD. A new view of pain as a homeostatic emotion. *Trends Neurosci* 2003;26(6):303-7. doi: 10.1016/s0166-2236(03)00123-1 [published Online First: 2003/06/12]
23. Wallden M, Sisson M. Modern disintegration and primal connectivity. *Journal of Bodywork and Movement Therapies* 2019;23(2):359-65. doi: 10.1016/j.jbmt.2019.03.003
24. McEwen BS. Protective and damaging effects of stress mediators: central role of the brain. *Dialogues Clin Neurosci* 2006;8(4):367-81. doi: 10.31887/DCNS.2006.8.4/bmcewen
25. Ellingsen DM, Napadow V, Protsenko E, et al. Brain Mechanisms of Anticipated Painful Movements and Their Modulation by Manual Therapy in Chronic Low Back Pain. *J Pain* 2018;19(11):1352-65. doi: 10.1016/j.jpain.2018.05.012 [published Online First: 20180703]
26. López-Martínez AE, Ramírez-Maestre C, Serrano-Ibáñez ER, et al. Intolerance of uncertainty moderates the relationship between catastrophizing, anxiety, and perceived pain in people with chronic nononcological pain. *Pain Med* 2022 doi: 10.1093/pm/pnac030 [published Online First: 20220216]
27. Parker R, Bergman E, Mntambo A, et al. Levels of physical activity in people with chronic pain. *S Afr J Physiother* 2017;73(1):323. doi: 10.4102/sajp.v73i1.323 [published Online First: 20170331]
28. McBeth J, Nicholl BI, Cordingley L, et al. Chronic widespread pain predicts physical inactivity: results from the prospective EPIFUND study. *European journal of pain (London, England)* 2010;14(9):972-9. doi: 10.1016/j.ejpain.2010.03.005 [published Online First: 2010/04/20]
29. Jones J, Rutledge DN, Jones KD, et al. Self-assessed physical function levels of women with fibromyalgia: a national survey. *Womens Health Issues* 2008;18(5):406-12. doi: 10.1016/j.whi.2008.04.005 [published Online First: 20080823]
30. Blitz B, Lowenthal M. The role of sensory restriction in problems with chronic pain. *J Chronic Dis* 1966;19(11):1119-25.

For peer review only - http://bmjopen.bmj.com/site/about/guidelines.xhtml

31. Mohan A, Vanneste S. Adaptive and maladaptive neural compensatory consequences of sensory deprivation-From a phantom percept perspective. *Prog Neurobiol* 2017;153:1-17. doi: 10.1016/j.pneurobio.2017.03.010 [published Online First: 20170411]
32. Tabor A, Burr C. Bayesian Learning Models of Pain: A Call to Action. *Current Opinion in Behavioral Sciences* 2019;26:54-61. doi: https://doi.org/10.1016/j.cobeha.2018.10.006
33. McParlin Z, Cerritelli F, Friston KJ, et al. Therapeutic Alliance as Active Inference: The Role of Therapeutic Touch and Synchrony. *Frontiers in Psychology* 2022;13 doi: 10.3389/fpsyg.2022.783694
34. Smith JA, Stabbert H, Bagwell JJ, et al. Do people with low back pain walk differently? A systematic review and meta-analysis. *J Sport Health Sci* 2022 doi: 10.1016/j.jshs.2022.02.001 [published Online First: 20220210]
35. Koch C, Hänsel F. Non-specific Low Back Pain and Postural Control During Quiet Standing-A Systematic Review. *Front Psychol* 2019;10:586. doi: 10.3389/fpsyg.2019.00586 [published Online First: 20190322]
36. Carrasco-Vega E, Ruiz-Muñoz M, Cuesta-Vargas A, et al. Individuals with fibromyalgia have a different gait pattern and a reduced walk functional capacity: a systematic review with meta-analysis. *PeerJ* 2022;10:e12908. doi: 10.7717/peerj.12908 [published Online First: 20220321]
37. Goossens N, Rummens S, Janssens L, et al. Association Between Sensorimotor Impairments and Functional Brain Changes in Patients With Low Back Pain: A Critical Review. *Am J Phys Med Rehabil* 2018;97(3):200-11. doi: 10.1097/phm.0000000000000859 [published Online First: 2017/11/08]
38. Cheng JC, Anzolin A, Berry M, et al. Dynamic Functional Brain Connectivity Underlying Temporal Summation of Pain in Fibromyalgia. *Arthritis Rheumatol* 2022;74(4):700-10. doi: 10.1002/art.42013 [published Online First: 20220310]
39. Karsan N, Goadsby PJ. Migraine Is More Than Just Headache: Is the Link to Chronic Fatigue and Mood Disorders Simply Due to Shared Biological Systems? *Front Hum Neurosci* 2021;15:646692. doi: 10.3389/fnhum.2021.646692 [published Online First: 20210603]
40. Chan AW, Tetzlaff JM, Altman DG, et al. SPIRIT 2013 statement: defining standard protocol items for clinical trials. *Ann Intern Med* 2013;158(3):200-7. doi: 10.7326/0003-4819-158-3-201302050-00583 [published Online First: 2013/01/09]
41. Schulz KF, Altman DG, Moher D. CONSORT 2010 statement: updated guidelines for reporting parallel group randomised trials. *BMJ (Clinical research ed)* 2010;340:c332. doi: 10.1136/bmj.c332 [published Online First: 2010/03/25]
42. Chan A-W, Tetzlaff JM, Gøtzsche PC, et al. SPIRIT 2013 explanation and elaboration: guidance for protocols of clinical trials. *BMJ : British Medical Journal* 2013;346:e7586. doi: 10.1136/bmj.e7586
43. Ghamkhar L, Kahlaee AH. Trunk muscles activation pattern during walking in subjects with and without chronic low back pain: a systematic review. *Pm r* 2015;7(5):519-26. doi: 10.1016/j.pmrj.2015.01.013 [published Online First: 20150126]
44. Gordt K, Gerhardy T, Najafi B, et al. Effects of Wearable Sensor-Based Balance and Gait Training on Balance, Gait, and Functional Performance in Healthy and Patient Populations: A Systematic Review and Meta-Analysis of Randomized

For peer review only - http://bmjopen.bmj.com/site/about/guidelines.xhtml

Controlled Trials. *Gerontology* 2018;64(1):74-89. doi: 10.1159/000481454 [published Online First: 20171101]

45. Borg G. Perceived exertion as an indicator of somatic stress. *Scand J Rehabil Med* 1970;2(2):92-8.

46. Huskisson EC. Measurement of pain. *Lancet* 1974;2:1127-31.

47. Taylor AM, Phillips K, Patel KV, et al. Assessment of physical function and participation in chronic pain clinical trials: IMMPACT/OMERACT recommendations. *Pain* 2016;157(9):1836-50. doi: 10.1097/j.pain.0000000000000577 [published Online First: 2016/04/09]

48. Brumagne S, Janssens L, Knapen S, et al. Persons with recurrent low back pain exhibit a rigid postural control strategy. *European spine journal : official publication of the European Spine Society, the European Spinal Deformity Society, and the European Section of the Cervical Spine Research Society* 2008;17(9):1177-84. doi: 10.1007/s00586-008-0709-7 [published Online First: 20080702]

49. Sullivan MJL, Bishop SR, Pivik J. The Pain Catastrophizing Scale: Development and validation. *Psychol Assess* 1995;7(4):524-32. doi: 10.1037/1040-3590.7.4.524

50. Bergstrom G, Jensen IB, Bodin L, et al. Reliability and factor structure of the Multidimensional Pain Inventory--Swedish Language Version (MPI-S). *Pain* 1998;75(1):101-10. [published Online First: 1998/04/16]

51. Turk DC, Rudy TE. Toward an empirically derived taxonomy of chronic pain patients: integration of psychological assessment data. *J Consult Clin Psychol* 1988;56(2):233-8. [published Online First: 1988/04/01]

52. Turk DC, Rudy TE. The robustness of an empirically derived taxonomy of chronic pain patients. *Pain* 1990;43(1):27-35. doi: 10.1016/0304-3959(90)90047-h [published Online First: 1990/10/01]

53. McKillop JM, Nielson WR. Improving the usefulness of the Multidimensional Pain Inventory. *Pain Res Manag* 2011;16(4):239-44. doi: 10.1155/2011/873424 [published Online First: 2011/11/08]

54. Gevins A, Cutillo B. Spatiotemporal dynamics of component processes in human working memory. *Electroencephalogr Clin Neurophysiol* 1993;87(3):128-43. doi: 10.1016/0013-4694(93)90119-g [published Online First: 1993/09/01]

55. Fang X, Liu C, Jiang Z. Reference values of gait using APDM movement monitoring inertial sensor system. *R Soc Open Sci* 2018;5(1):170818. doi: 10.1098/rsos.170818 [published Online First: 2018/02/08]

56. Hermens HJ, Frerik B. The SENIAM project (Surface ElectroMyoGraphy for the Non-Invasive Assessment of Muscles) 2020 [cited 2020 November 26]. Available from: http://www.seniam.org/.

57. Hermens HJ, Freriks B, Disselhorst-Klug C, et al. Development of recommendations for SEMG sensors and sensor placement procedures. *Journal of electromyography and kinesiology : official journal of the International Society of Electrophysiological Kinesiology* 2000;10(5):361-74. doi: 10.1016/s1050-6411(00)00027-4 [published Online First: 2000/10/06]

58. Kribus-Shmiel L, Zeilig G, Sokolovski B, et al. How many strides are required for a reliable estimation of temporal gait parameters? Implementation of a new algorithm on the phase coordination index. *PLoS One* 2018;13(2):e0192049. doi: 10.1371/journal.pone.0192049 [published Online First: 2018/02/09]

59. Cohen J. Statistical power analysis for the behavioral sciences. 2nd ed. Hillsdale, N.J.: L. Erlbaum Associates 1988.

For peer review only - http://bmjopen.bmj.com/site/about/guidelines.xhtml

60. Tzagarakis G, Tsivgoulis S, Papagelopoulos P, et al. Influence of Acute Anterior Cruciate Ligament Deficiency in Gait Variability. *Journal of International Medical Research* 2010;38(2):511-25. doi: 10.1177/147323001003800214
61. Harbourne RT, Stergiou N. Movement variability and the use of nonlinear tools: principles to guide physical therapist practice. *Phys Ther* 2009;89(3):267-82. doi: 10.2522/ptj.20080130 [published Online First: 20090123]
62. Strongman C, Morrison A. A scoping review of non-linear analysis approaches measuring variability in gait due to lower body injury or dysfunction. *Human Movement Science* 2020;69:102562. doi: https://doi.org/10.1016/j.humov.2019.102562
63. Molander P, Dong HJ, Äng B, et al. The role of pain in chronic pain patients' perception of health-related quality of life: a cross-sectional SQRP study of 40,000 patients. *Scand J Pain* 2018;18(3):417-29. doi: 10.1515/sjpain-2018-0003
64. Rice D, Nijs J, Kosek E, et al. Exercise-Induced Hypoalgesia in Pain-Free and Chronic Pain Populations: State of the Art and Future Directions. *J Pain* 2019;20(11):1249-66. doi: 10.1016/j.jpain.2019.03.005 [published Online First: 20190321]
65. van Hecke O, Torrance N, Smith BH. Chronic pain epidemiology - where do lifestyle factors fit in? *Br J Pain* 2013;7(4):209-17. doi: 10.1177/2049463713493264

For peer review only - http://bmjopen.bmj.com/site/about/guidelines.xhtml

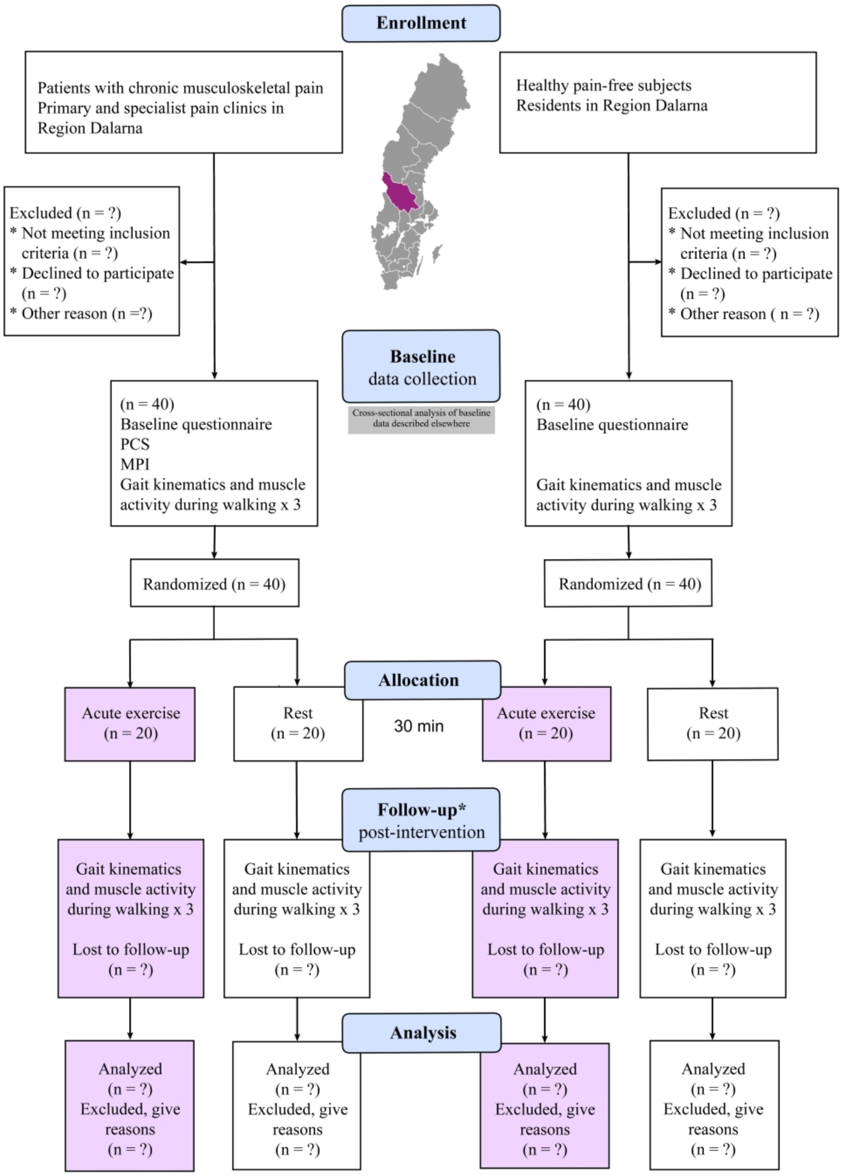

Flowchart

1147x1574mm (96 x 96 DPI)

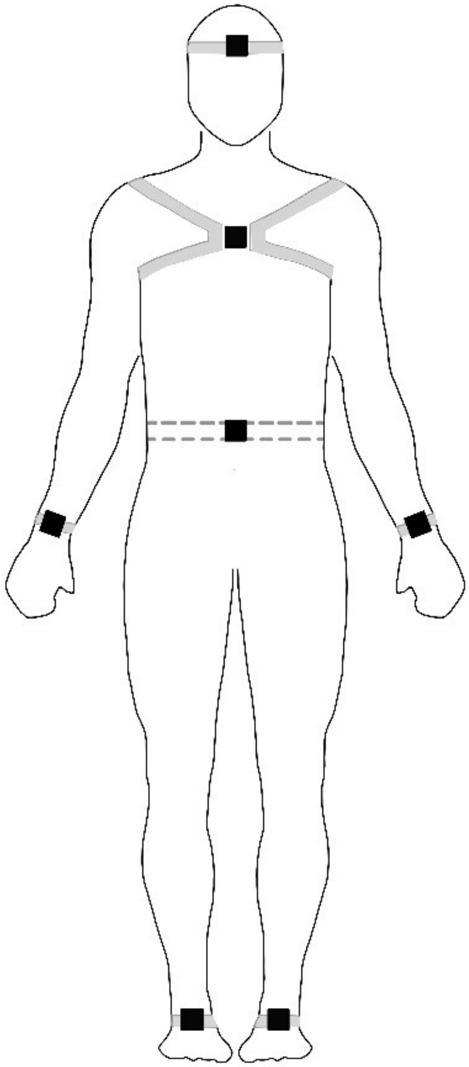

IMU Sensor placement

79x112mm (220 x 220 DPI)

Muscle	EMG electrode locations	Orientation	Illustration
Erector Spinae Neck, bilateral	Placed 20 mm lateral to the median line at the level of C4-C5 spinous process	Vertical	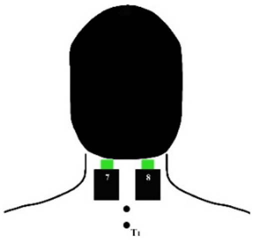
Erector Spinae Longissimus (EL), bilateral	Placed 2 finger widths lateral from the spinous process of L1.	Vertical	
Erector Spinae Iliocostalis (IL), bilateral	Placed 1 finger width medial from a line from the posterior spina iliaca superior to the lowest point of the lower rib, at the level of L2.	Vertical	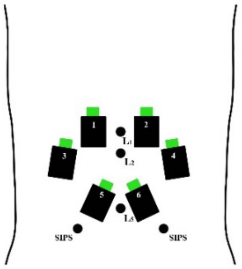
Multifidus (MF), bilateral	Placed on and aligned with a line from caudal tip posterior spina iliaca superior to the interspace between L1 and L2 interspace at the level of L5 spinous process (i.e. about 20-30 mm from midline).	Aligned with line, 45° from horizontal	

EMG, electromyography

EMG Sensor placement

390x275mm (79 x 87 DPI)

STANDARD PROTOCOL ITEMS: RECOMMENDATIONS FOR INTERVENTIONAL TRIALS

SPIRIT 2013 Checklist: Recommended items to address in a clinical trial protocol and related documents by Westergren et al.

Section/item	Item No	Description	Addressed on page number
**Administrative information**			
Title	1	Descriptive title identifying the study design, population, interventions, and, if applicable, trial acronym	1
Trial registration	2a	Trial identifier and registry name. If not yet registered, name of intended registry	4
	2b	All items from the World Health Organization Trial Registration Data Set	4
Protocol version	3	Date and version identifier	5
Funding	4	Sources and types of financial, material, and other support	22
Roles and responsibilities	5a	Names, affiliations, and roles of protocol contributors	1, 22
	5b	Name and contact information for the trial sponsor	22
	5c	Role of study sponsor and funders, if any, in study design; collection, management, analysis, and interpretation of data; writing of the report; and the decision to submit the report for publication, including whether they will have ultimate authority over any of these activities	22
	5d	Composition, roles, and responsibilities of the coordinating centre, steering committee, endpoint adjudication committee, data management team, and other individuals or groups overseeing the trial, if applicable (see Item 21a for data monitoring committee)	Not applicable

For peer review only

**Introduction**			
Background and rationale	6a	Description of research question and justification for undertaking the trial, including summary of relevant studies (published and unpublished) examining benefits and harms for each intervention	6-8
	6b	Explanation for choice of comparators	8-9
Objectives	7	Specific objectives or hypotheses	9-10
Trial design	8	Description of trial design including type of trial (eg, parallel group, crossover, factorial, single group), allocation ratio, and framework (eg, superiority, equivalence, noninferiority, exploratory)	9-10
**Methods: Participants, interventions, and outcomes**			
Study setting	9	Description of study settings (eg, community clinic, academic hospital) and list of countries where data will be collected. Reference to where list of study sites can be obtained	9
Eligibility criteria	10	Inclusion and exclusion criteria for participants. If applicable, eligibility criteria for study centres and individuals who will perform the interventions (eg, surgeons, psychotherapists)	11
Interventions	11a	Interventions for each group with sufficient detail to allow replication, including how and when they will be administered	11-12
	11b	Criteria for discontinuing or modifying allocated interventions for a given trial participant (eg, drug dose change in response to harms, participant request, or improving/worsening disease)	17
	11c	Strategies to improve adherence to intervention protocols, and any procedures for monitoring adherence (eg, drug tablet return, laboratory tests)	Not Applicable
	11d	Relevant concomitant care and interventions that are permitted or prohibited during the trial	Not Applicable
Outcomes	12	Primary, secondary, and other outcomes, including the specific measurement variable (eg, systolic blood pressure), analysis metric (eg, change from baseline, final value, time to event), method of aggregation (eg, median, proportion), and time point for each outcome. Explanation of the clinical relevance of chosen efficacy and harm outcomes is strongly recommended	12-13
Participant timeline	13	Time schedule of enrolment, interventions (including any run-ins and washouts), assessments, and visits for participants. A schematic diagram is highly recommended (see Figure)	13-16, Figure 1 Table 1

For peer review only - http://bmjopen.bmj.com/site/about/guidelines.xhtml

Sample size	14	Estimated number of participants needed to achieve study objectives and how it was determined, including clinical and statistical assumptions supporting any sample size calculations	10
Recruitment	15	Strategies for achieving adequate participant enrolment to reach target sample size	10-11

Methods: Assignment of interventions (for controlled trials)

Allocation:

Sequence generation	16a	Method of generating the allocation sequence (eg, computer-generated random numbers), and list of any factors for stratification. To reduce predictability of a random sequence, details of any planned restriction (eg, blocking) should be provided in a separate document that is unavailable to those who enrol participants or assign interventions	11-12
Allocation concealment mechanism	16b	Mechanism of implementing the allocation sequence (eg, central telephone; sequentially numbered, opaque, sealed envelopes), describing any steps to conceal the sequence until interventions are assigned	11-12
Implementation	16c	Who will generate the allocation sequence, who will enrol participants, and who will assign participants to interventions	10-12
Blinding (masking)	17a	Who will be blinded after assignment to interventions (eg, trial participants, care providers, outcome assessors, data analysts), and how	11
	17b	If blinded, circumstances under which unblinding is permissible, and procedure for revealing a participant's allocated intervention during the trial	Not Applicable

Methods: Data collection, management, and analysis

Data collection methods	18a	Plans for assessment and collection of outcome, baseline, and other trial data, including any related processes to promote data quality (eg, duplicate measurements, training of assessors) and a description of study instruments (eg, questionnaires, laboratory tests) along with their reliability and validity, if known. Reference to where data collection forms can be found, if not in the protocol	12-13<br>15-17<br>Table 1
	18b	Plans to promote participant retention and complete follow-up, including list of any outcome data to be collected for participants who discontinue or deviate from intervention protocols	Not applicable

Data management	19	Plans for data entry, coding, security, and storage, including any related processes to promote data quality (eg, double data entry; range checks for data values). Reference to where details of data management procedures can be found, if not in the protocol	17
Statistical methods	20a	Statistical methods for analysing primary and secondary outcomes. Reference to where other details of the statistical analysis plan can be found, if not in the protocol	18
	20b	Methods for any additional analyses (eg, subgroup and adjusted analyses)	18
	20c	Definition of analysis population relating to protocol non-adherence (eg, as randomised analysis), and any statistical methods to handle missing data (eg, multiple imputation)	18
**Methods: Monitoring**			
Data monitoring	21a	Composition of data monitoring committee (DMC); summary of its role and reporting structure; statement of whether it is independent from the sponsor and competing interests; and reference to where further details about its charter can be found, if not in the protocol. Alternatively, an explanation of why a DMC is not needed	17
	21b	Description of any interim analyses and stopping guidelines, including who will have access to these interim results and make the final decision to terminate the trial	Not applicable
Harms	22	Plans for collecting, assessing, reporting, and managing solicited and spontaneously reported adverse events and other unintended effects of trial interventions or trial conduct	17
Auditing	23	Frequency and procedures for auditing trial conduct, if any, and whether the process will be independent from investigators and the sponsor	Not applicable
**Ethics and dissemination**			
Research ethics approval	24	Plans for seeking research ethics committee/institutional review board (REC/IRB) approval	4,18
Protocol amendments	25	Plans for communicating important protocol modifications (eg, changes to eligibility criteria, outcomes, analyses) to relevant parties (eg, investigators, REC/IRBs, trial participants, trial registries, journals, regulators)	Not applicable

Consent or assent	26a	Who will obtain informed consent or assent from potential trial participants or authorised surrogates, and how (see Item 32)	15
	26b	Additional consent provisions for collection and use of participant data and biological specimens in ancillary studies, if applicable	Not applicable
Confidentiality	27	How personal information about potential and enrolled participants will be collected, shared, and maintained in order to protect confidentiality before, during, and after the trial	17
Declaration of interests	28	Financial and other competing interests for principal investigators for the overall trial and each study site	22
Access to data	29	Statement of who will have access to the final trial dataset, and disclosure of contractual agreements that limit such access for investigators	17
Ancillary and post-trial care	30	Provisions, if any, for ancillary and post-trial care, and for compensation to those who suffer harm from trial participation	17
Dissemination policy	31a	Plans for investigators and sponsor to communicate trial results to participants, healthcare professionals, the public, and other relevant groups (eg, via publication, reporting in results databases, or other data sharing arrangements), including any publication restrictions	18
	31b	Authorship eligibility guidelines and any intended use of professional writers	22
	31c	Plans, if any, for granting public access to the full protocol, participant-level dataset, and statistical code	22-23
**Appendices**			
Informed consent materials	32	Model consent form and other related documentation given to participants and authorised surrogates	15
Biological specimens	33	Plans for collection, laboratory evaluation, and storage of biological specimens for genetic or molecular analysis in the current trial and for future use in ancillary studies, if applicable	Not applicable

*It is strongly recommended that this checklist be read in conjunction with the SPIRIT 2013 Explanation & Elaboration for important clarification on the items. Amendments to the protocol should be tracked and dated. The SPIRIT checklist is copyrighted by the SPIRIT Group under the Creative Commons "Attribution-NonCommercial-NoDerivs 3.0 Unported" license.

For peer review only

HÖGSKOLAN DALARNA

KOD ___________

Samtycke till att delta i projektet

Jag har fått muntlig och skriftlig informationen om projektet och har haft möjlighet att ställa frågor. Jag får behålla den skriftliga informationen.

☐ Jag samtycker till att delta i projektet *Fysisk aktivitet, rörelsekontroll och muskelaktivitet hos personer med långvarig smärta.*

☐ Jag samtycker till att uppgifter om mig behandlas på det sätt som beskrivs i forskningspersonsinformationen.

Ort och datum	Underskrift

Högskolan Dalarna EPN-nr 2018-307

S 791 88 Falun Tfn Fax www.du.se

Sweden +46 23-77 80 00 +46 23-77 80 80

For peer review only - http://bmjopen.bmj.com/site/about/guidelines.xhtml

HÖGSKOLAN DALARNA

KOD ___________

Forskningspersonens kopia

Samtycke till att delta i projektet

Jag har fått muntlig och skriftlig informationen om projektet och har haft möjlighet att ställa frågor. Jag får behålla den skriftliga informationen.

☐ Jag samtycker till att delta i projektet *Fysisk aktivitet, rörelsekontroll och muskelaktivitet hos personer med långvarig smärta.*

☐ Jag samtycker till att uppgifter om mig behandlas på det sätt som beskrivs i forskningspersonsinformationen.

Ort och datum	Underskrift

For peer review only

For peer review only - http://bmjopen.bmj.com/site/about/guidelines.xhtml

FYSISK AKTIVITET VID LÅNGVARIG SMÄRTA

HÖGSKOLAN DALARNA

Vill du vara med i en forskningsstudie?

Projektet har som syfte att förbättra rehabiliteringen för personer med långvarig smärta.

Långvarig smärta i leder och muskler leder till fysisk och psykisk ohälsa. En viktig del av behandlingen är fysisk träning som anpassas till individens förutsättningar.

Moderna aktivitetsmätare som ser ut som vanliga armbandsklockor blir alltmer vanliga i forskning och utveckling som rör fysisk aktivitet och träning för olika patientgrupper, men få sådana studier har genomförts bland personer med långvarig smärta.

Befintlig forskning har visat att en del personer som lever med långvarig smärta har förändrat rörelsemönster och muskelaktivering, men återigen finns det få studier visar hur fysisk aktivitet och träning påverkar sådana avvikande rörelsebeteenden. För att få sådan vetenskaplig kunskap behövs det mätningar på både personer som lever med långvarig smärta men även mätningar på friska smärtfria personer.

Vad innebär det att delta i projektet?

Du kommer först att bli kontaktad via telefon där du får mer information om projektet. Om du då är intresserad att delta som forskningsperson och matchar projektets urvalskriterier (ålder, typ av smärta med mera), bokas ett besök in vid Högskolan Dalarnas idrotts- och hälsolaboratorium på Lugnet i Falun.

Under besöket som beräknas ta cirka tre timmar får du:

1. Besvara två frågeformulär med frågor om ditt nuvarande hälsotillstånd, din bakgrund (ålder, yrke etc.) och frågor som berör smärta.
2. Bära flera mindre mätare som registrerar rörelsemönster, muskelaktivitet och fysisk aktivitet. Du kommer gå en kortare sträcka inomhus under några minuter på tre olika sätt och därefter lottas till att antingen cykla eller vila i 30 minuter. Därefter går du samma sträcka som tidigare. Avslutningsvis kommer du att promenera i 18 minuter på ett gångband samtidigt som din utandningsluft mäts via en ansiktsmask.
3. Efter testdagen får du med dig tre mätare hem för att bära under tre dagar på handleden och vid midjan. Dessa postar du åter i ett frankerat kuvert.

Vad händer med mina uppgifter?

Högskolan Dalarna är forskningshuvudman. All insamlad data kommer att ligga till grund för vetenskapliga publikationer. Datan kommer att kodas och förvaras på Högskolan Dalarnas lösenordskyddade dataserver.

Kontakt

Jens Westergren, Doktorand, Vårdvetenskap, e-post: jws@du.se, telefon: 023-77 80 69
Veronica Sjöberg, Doktorand, Vårdvetenskap, e-post: vsj@du.se , telefon: 023-77 87 57

MER INFORMATION OM PROJEKTET HITTAR DU PÅ DU.SE/PAIN

du.se

For peer review only - http://bmjopen.bmj.com/site/about/guidelines.xhtml
